# Human lifespan and sex-specific patterns of resilience to disease: a retrospective population-wide cohort study

**DOI:** 10.1186/s12916-023-03206-w

**Published:** 2024-01-08

**Authors:** Joaquim Sol, Marta Ortega-Bravo, Manuel Portero-Otín, Gerard Piñol-Ripoll, Vicent Ribas-Ripoll, Eva Artigues-Barberà, Miquel Butí, Reinald Pamplona, Mariona Jové

**Affiliations:** 1 Catalan Health Institute (ICS), Lleida Research Support Unit (USR), Fundació Institut Universitari per a la Recerca en Atenció Primària de Salut Jordi Gol i Gurina (IDIAP JGol), Lleida, Spain; 2 Department of Experimental Medicine, University of Lleida-Lleida Biomedical Research Institute (UdL-IRBLleida), Lleida, Spain; 3 Unitat Trastorns Cognitius, Clinical Neuroscience Research, Santa Maria University Hospital, IRBLleida, Lleida, Spain; 4 Eurecat, Centre Tecnològic de Catalunya, Barcelona, Spain

**Keywords:** Human lifespan, Health span, Primary care, Electronic health records, Aging, Age-related diseases, Escapers

## Abstract

**Background:**

Slower paces of aging are related to lower risk of developing diseases and premature death. Therefore, the greatest challenge of modern societies is to ensure that the increase in lifespan is accompanied by an increase in health span. To better understand the differences in human lifespan, new insight concerning the relationship between lifespan and the age of onset of diseases, and the ability to avoid them is needed. We aimed to comprehensively study, at a population-wide level, the sex-specific disease patterns associated with human lifespan.

**Methods:**

Observational data from the SIDIAP database of a cohort of 482,058 individuals that died in Catalonia (Spain) at ages over 50 years old between the 1st of January 2006 and the 30th of June 2022 were included. The time to the onset of the first disease in multiple organ systems, the prevalence of escapers, the percentage of life free of disease, and their relationship with lifespan were evaluated considering sex-specific traits.

**Results:**

In the study cohort, 50.4% of the participants were women and the mean lifespan was 83 years. The results show novel relationships between the age of onset of disease, health span, and lifespan. The key findings include: Firstly, the onset of both single and multisystem diseases is progressively delayed as lifespan increases. Secondly, the prevalence of escapers is lower in lifespans around life expectancy. Thirdly, the number of disease-free systems decreases until individuals reach lifespans around 87–88 years old, at which point it starts to increase. Furthermore, long-lived women are less susceptible to multisystem diseases. The associations between health span and lifespan are system-dependent, and disease onset and the percentage of life spent free of disease at the time of death contribute to explaining lifespan variability. Lastly, the study highlights significant system-specific disparities between women and men.

**Conclusions:**

Health interventions focused on delaying aging and age-related diseases should be the most effective in increasing not only lifespan but also health span. The findings of this research highlight the relevance of Electronic Health Records in studying the aging process and open up new possibilities in age-related disease prevention that should assist primary care professionals in devising individualized care and treatment plans.

**Supplementary Information:**

The online version contains supplementary material available at 10.1186/s12916-023-03206-w.

## Background

Aging is the main risk factor for developing highly prevalent diseases including cancer, cardiovascular diseases, diabetes mellitus, neurodegenerative diseases, and chronic respiratory diseases, among others [1]. By 2050, the number of people aged 65 or older in Europe and the USA will have increased from 20 to 25% [2], and two theories have been put forward to address the effect of this increase in lifespan on the prevalence of diseases and disability of the population. The “compression of morbidity” theory claims that the onset of morbidity is postponed with the increase in lifespan, resulting in an extended health span [3]. In contrast, the “expansion of morbidity” theory suggests that health span is not increased, resulting in additional years of life accompanied by morbidity and disability [4]. However, to discern the interplay among lifespan, health span and morbidity, further insight, at the individual level, of the relationship between lifespan and the age of onset of the diseases and the ability to avoid them is needed. Apart from these two opposing theories, a more recent individual-centered theory suggests that there is a continuum of individual paces of aging (from accelerated to deaccelerated) that results in differing onset of symptoms and diseases depending on individual vulnerability [5].

The aging process affects all organs and tissues, leading to changes like altered pancreatic function [6], shifts in adipose tissue distribution [7], loss of cognitive and motor abilities in the nervous system [8], circulatory system issues [9], reduced respiratory capacity [10], liver volume and blood flow decline [11], diminished nutrient absorption in the gastrointestinal tract [12], skeletal muscle mass and functionality reduction [13], and kidney structural alterations [14]. It has previously been reported that the inheritable factor of human lifespan could explain from 20 to 40% of the variability (with the remaining being related to environmental traits) and that extreme lifespan clusters in specific families [15], leaving room for modulating external factors to increase lifespan. Previous studies [16–18] reported that although the changes related to aging are common in all persons, the speed of loss of organ and tissue functionality differs between individuals, even at early ages, implying that aging has an inter-individual effect and remarking the differences between chronological and biological age. Specifically, the Dunedin study, carried out in a population of 869 young adults followed up from birth to age 45, showed that individuals who aged more rapidly according to their biological age were less physically able, had greater cognitive decline and brain aging, and presented worse self-perceived health [16, 17]. The objective of this study was to characterize biological aging in the early stages of adulthood and the relationship between the individual aging process and the development of chronic diseases. Death in this study population is yet to be determined. Similarly, the Baltimore Longitudinal Study of Aging [19] revealed, in a smaller cohort (968 participants) that slower paces of aging were related to better health status and lower risk of death. In this line, a study from the UK Biobank [20] calculated biological age specifically for each organ system and used this to predict the development of chronic diseases and premature death with high accuracy. However, the maximum lifespan of the participants in this study was 83 years, and extreme lifespan was not assessed. Centenarians and supercentenarians from the New England Centenarians Study showed that the prevalence of escapers (defined as individuals with onset of the first studied disease after age 100 years) was higher in centenarians, semisupercentenarians, and supercentenarians, compared with non-centenarians [21]. In this case, participants who died before 90 were considered as a single group, and shorter lifespans were not addressed. Other projects such as the Global Burden of Disease Study [22] investigated death and disability across all ages but either focused only on the causes of death or on chronological age and population alive at each age rather than the biological age or lifespan of the participants. In this sense, evaluating the interplay between longevity and diseases, including all the range of possible lifespans in a population-wide cohort should be crucial to discern the patterns that define human lifespan.

Until recently, the main increase in lifespan was related to progress in the prevention and treatment of infectious diseases (COVID-19 aside), but this tendency has now shifted and this increase is attributable to the improvement in chronic disease care and the decline in death rates after 65 [23]. In this sense, the characterization of age-related diseases (onset, progression, time between diagnosis and death) in large populations and the definition of the diseases more implicated in the human lifespan are crucial to continue delaying their onset. This knowledge should contribute to defining health strategies to decrease the pace of aging, promote P4 (predictive, preventive, personalized, and participatory) medicine, and increase lifespan and health span.

In the present study, we offer a comprehensive study, at a population-wide level, of the sex-specific pathological patterns, age of onset, multisystem involvement, and years free of disease and their association with human lifespan. We evaluated, in a cohort of 482,058 participants (50.4% women) with a mean lifespan of 83 years (ranging from 50 to 112), survival models for the onset of disease in multiple organ systems, the ability to avoid disease, and the percentage of life free of disease, all of which were evaluated according to the lifespan of the individuals. These results were specifically evaluated for each sex, considering the organ systems independently and accounting for multisystem involvement. The results show novel relationships among the age of onset of disease, health span, lifespan, and how these are adapted in escapers, showing system-specific and disease-specific trends that are highly relevant in explaining lifespan variability. Furthermore, there are notable differences between women and men at the system level in the determination of health span.

## Methods

### Design, data source, and study setting

We analyzed data obtained from the Information System for the Development of Research in Primary Care (SIDIAP) in the autonomous community of Catalonia (Spain). The SIDIAP database has collected anonymized electronic health records (EHR) data since 2005 of more than 5 million people, which represents 80% of the Catalan population and is representative of the region as a whole [24]. This manuscript has not been prepared in collaboration with SIDIAP and therefore does not necessarily reflect their opinions or points of view. The quality and accuracy are the sole responsibility of the authors. The authors had access only to the cleaned tables provided by SIDIAP.

The longitudinal cohort study considers all men over 43 years old and women over 48 years old at the 1^st^ of January 2006 included in SIDIAP, with at least two laboratory blood results during the first 7 years of follow-up, that died between the 1st of January 2006 and the 30th of June 2022 at an age of 50 years or older. The follow-up time for the participants included their entire life. Data collected for this project included the sex specified in the clinical history of the individuals, date of birth and death, diagnoses made in primary care, and date of diagnosis. The SIDIAP database included 8,265,343 individuals. Among those, 482,058 individuals met the inclusion criteria and were analyzed (Additional file 1).

### Diagnoses and classification

We selected those age-related chronic conditions that caused the death of more than 1% of the population after 50 years of age [22] together with relevant causes of frailty [25] from 8 of the main categories (29 subcategories) of the International Classification of Diseases, Tenth Revision (ICD-10). The list of the codes and diseases included can be found in Additional file 2. The classifications take into account the main body system affected. These include neoplasms, endocrine, nutritional, and metabolic diseases, diseases of the nervous system, diseases of the circulatory system, diseases of the respiratory system, diseases of the digestive system, diseases of the musculoskeletal system and connective tissue, and diseases of the genitourinary system. Diagnoses after the 1st of January 2005 were directly introduced in the EHR. Prior diagnoses were collected in paper records and were introduced into the EHR during the virtualization of the clinical history. Diagnoses with date of diagnosis previous to date of birth were excluded.

### Statistical analyses

Participants were stratified according to their sex and lifespan (from 50 years old to 105+). Participants who died after 105 years old (*n* = 450) were included in the 105+ group. In the tables, we grouped the data by decades of death to simplify the information.

First, we performed survival analyses to evaluate the hazard ratios for the onset of the different diseases. We made Kaplan–Meier curves and Cox proportional hazards regressions using the lifespan (age of death), sex, and the interaction of the two as predictors. Results were expressed as hazard ratios (HR) with the respective 95% confidence intervals. To assess hazard ratios for multisystem involvement, we used the same approach evaluating the time to onset of a specific number of diseases from different body systems (from 2 to 8). Data regarding numbers at risk can be found in the Additional file 3. Second, the ability to escape disease was assessed. The prevalence of escapers (individuals that died without a specific disease) and its evolution across their lifespan were evaluated. For this purpose, the prevalence of escapers was computed, and locally estimated scatterplot smoothing (LOESS) curves of the second degree and span = 0.75, which are the default values of the ggplot package, were adjusted using the lifespan as a predictor variable of the prevalence. To account for multisystem involvement, the same approach was used to evaluate the number of systems free of disease. Third, health span was evaluated in those individuals with disease. The median percentage of life free of disease was computed at each lifespan, and LOESS curves of the second degree and span = 0.75 were made. Similar body system patterns of evolution of the prevalence of escapers and percentage of life free of disease were grouped using a k-means clustering algorithm. The optimal number of clusters was selected as the number of clusters with higher average silhouette width for a number of clusters from 2 to 7. Fourth, the importance of the diseases and the percentage of life free of disease in explaining lifespan was evaluated. A multiple factor analysis (MFA) in the sense of Escofier-Pagès [26] was performed including these variables as active variables, and the corresponding individual scores and variable loadings were computed. Specifically, sex and escaping the disease (yes or no) for each ICD-10 category were included as categorical variables, and the number of systems free of disease and the percentage of life free of disease for each ICD-10 category were included as numerical variables. The analyses were performed using R version 4.0.2, employing the survival, survminer, FactoMineR, and factoextra packages. A sub-analysis including only individuals that died up to 30th of June 2019 was performed to assess the effect of the COVID-19 pandemic. Results were consistent with those from the main analysis (data not shown).

## Results

### Population of study

The study sample included 482,058 participants (50.4% women) with a mean lifespan of 83 years, ranging from 50 to 112. Lifestyle, clinical, and socioeconomic variables of the study population for each decade of death are described in Table 1.

**Table 1:** Description of the population according to the death decade

**Variable**	**All (*N* = 482,058)**	**50–59 (*N* = 15,552)**	**60–69 (*N* = 43,872)**	**70–79 (*N* = 95,463)**	**80–89 (*N* = 200,723)**	**90–99 (*N* = 120,169)**	**100–105 + (*N* = 6279)**
Sex							
Women	243,127 (50.4%)	5224 (33.6%)	13,648 (31.1%)	35,106 (36.8%)	103,706 (51.7%)	80,457 (67.0%)	4986 (79.4%)
Men	238,931 (49.6%)	10,328 (66.4%)	30,224 (68.9%)	60,357 (63.2%)	97,017 (48.3%)	39,712 (33.0%)	1293 (20.6%)
Lifestyle variables							
Smoking status							
Non-smoker	248,756 (54.2%)	3864 (26.8%)	14,093 (33.5%)	41,028 (44.8%)	110,481 (57.8%)	75,278 (66.3%)	4012 (70.5%)
Smoker	55,660 (12.1%)	6291 (43.6%)	12,360 (29.4%)	14,363 (15.7%)	16,173 (8.46%)	6174 (5.43%)	299 (5.25%)
Ex-smoker	154,293 (33.6%)	4275 (29.6%)	15,614 (37.1%)	36,287 (39.6%)	64,589 (33.8%)	32,148 (28.3%)	1380 (24.2%)
Alcohol consumption							
Non-drinker	332,014 (78.6%)	7656 (61.3%)	23,969 (62.0%)	59,531 (70.5%)	142,630 (81.0%)	93,426 (88.4%)	4802 (92.9%)
Low risk	84,102 (19.9%)	3785 (30.3%)	12,696 (32.9%)	23,055 (27.3%)	32,220 (18.3%)	11,982 (11.3%)	364 (7.04%)
High risk	6260 (1.48%)	1055 (8.44%)	1981 (5.13%)	1812 (2.15%)	1155 (0.66%)	252 (0.24%)	5 (0.10%)
Clinical variables							
Systolic blood pressure	130 [116;139]	127 [115;137]	130 [117;138]	130 [118;139]	130 [116;139]	128 [115;139]	127 [114;138]
Diastolic blood pressure	70.0 [62.0;79.0]	78.0 [70.0;84.0]	75.0 [68.0;81.0]	71.0 [64.0;80.0]	70.0 [61.0;78.0]	70.0 [60.0;77.0]	70.0 [60.0;76.0]
Body mass index	26.8 [23.7;30.2]	27.1 [23.6;31.4]	27.6 [24.2;31.5]	27.5 [24.3;31.0]	26.8 [23.8;30.1]	25.8 [22.9;29.1]	24.8 [21.9;27.9]
Socioeconomic variables							
Quintile of MEDEA index							
0 rural	46,916 (9.73%)	1363 (8.76%)	3704 (8.44%)	8253 (8.65%)	20,189 (10.1%)	12,854 (10.7%)	553 (8.81%)
1 rural	35,823 (7.43%)	1185 (7.62%)	3030 (6.91%)	6444 (6.75%)	15,253 (7.60%)	9427 (7.84%)	484 (7.71%)
1 urban	93,729 (19.4%)	2501 (16.1%)	7350 (16.8%)	16,449 (17.2%)	38,608 (19.2%)	27,108 (22.6%)	1713 (27.3%)
2 rural	64,191 (13.3%)	2044 (13.1%)	5519 (12.6%)	11,804 (12.4%)	27,095 (13.5%)	16,890 (14.1%)	839 (13.4%)
2 urban	69,881 (14.5%)	2280 (14.7%)	6361 (14.5%)	13,734 (14.4%)	28,921 (14.4%)	17,628 (14.7%)	957 (15.2%)
3 urban	91,806 (19.0%)	3192 (20.5%)	9098 (20.7%)	19,722 (20.7%)	37,987 (18.9%)	20,828 (17.3%)	979 (15.6%)
4 urban	79,709 (16.5%)	2987 (19.2%)	8810 (20.1%)	19,056 (20.0%)	32,670 (16.3%)	15,432 (12.8%)	754 (12.0%)

The reported values are the last registered values in SIDIAP. MEDEA: Index of mortality in small Spanish areas and socioeconomic and environmental inequalities. Alcohol consumption of high risk corresponds to usual consumption of 17 standard drinks or more per week in women, 28 or more in men; to sporadic consumption, at least once per month, of 5 or more standard drinks in women, 6 or more in men; to consumption under 16 years old, when using dangerous machinery, when taking medication that interacts with alcohol or when pregnant. MEDEA index is based on [27]; higher values indicate more socioeconomic deprivation of the primary health center to which the patient is assigned

### Disease incidence

**Fig. 1:**
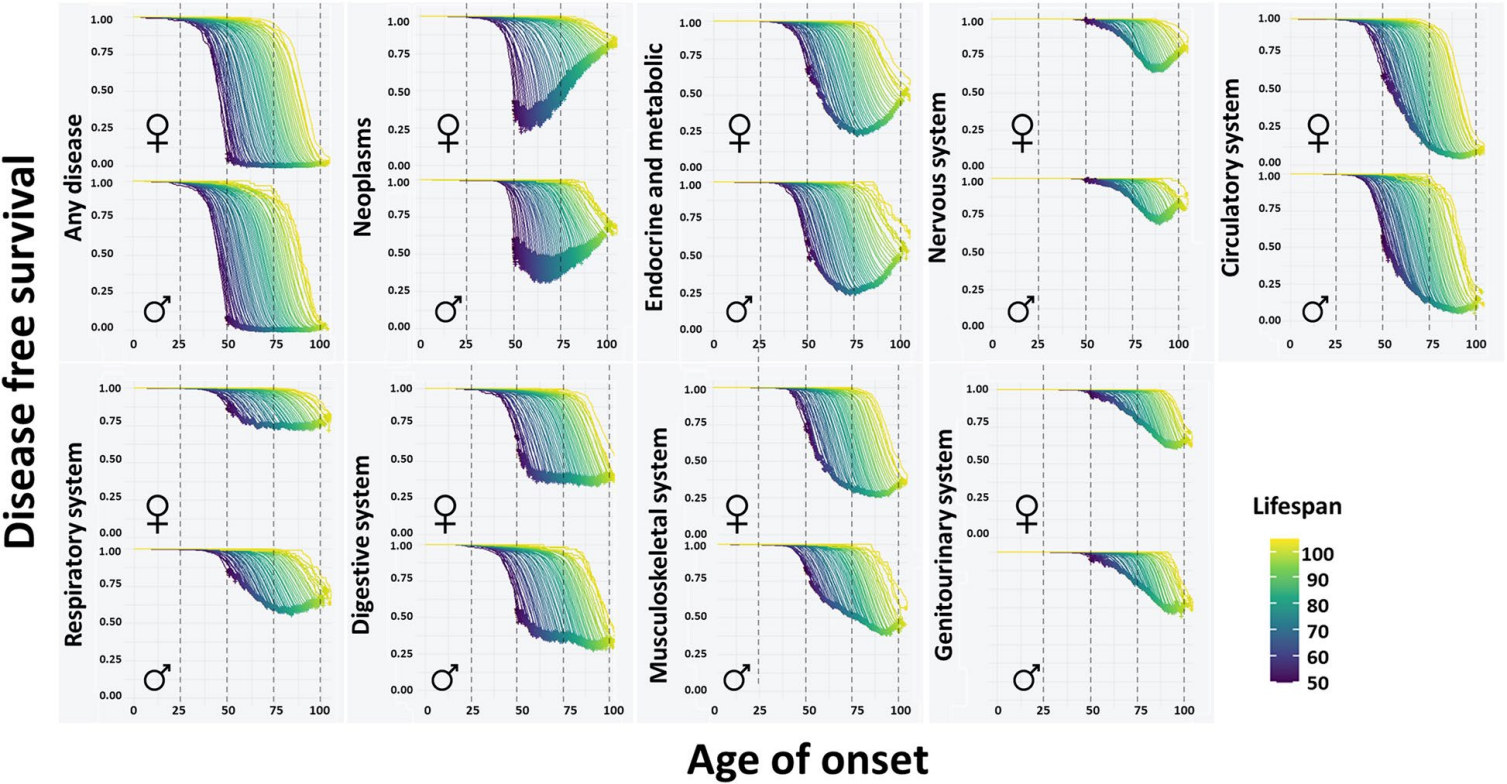
Kaplan-Meier curves for the onset of age-related diseases in different biological systems according to age of death, stratified by sex. The proportion of individuals free of disease (*y*-axis) at each age (*x*-axis) is represented stratifying by lifespan of individuals and sex. Each panel includes the curves from both sexes corresponding to the same system. Curves that decay earlier indicate a higher risk of an early onset of the disease. Age of onset is considered as the age of diagnosis of the first disease in the system. The specific diseases belonging to each category can be found in Additional file 2

First, a survival analysis was performed to evaluate the age of onset of the first disease from each main ICD-10 category according to sex and lifespan. The results displayed a continuous spectrum of Kaplan–Meier curves in all the systems studied, which were delayed over time as the lifespan increased (Fig. 1). In some cases, the curves were different for each sex (neoplasms, diseases of the respiratory system, and diseases of the musculoskeletal system). When analyzing the Cox regressions by lifespan and sex, we found HR between 0.85 and 0.90 for each increased year of lifespan for all the ICD-10 categories and for the onset of the first disease (Table 2), indicating that the most long-lived individuals had much longer health spans. Regarding sex, hazard rates for neoplasms and for diseases of the respiratory system were remarkably lower in women (HR of 0.72 and 0.58, respectively), whereas hazard rates for diseases of the nervous system and for diseases of the musculoskeletal system and connective tissue were higher (HR of 1.34 and 1.64, respectively). Small but relevant interactions between lifespan and sex were found, mainly in neoplasms and endocrine, nutritional, and metabolic diseases, resulting in a lower hazard for neoplasms and a higher hazard for endocrine, nutritional, and metabolic diseases in shorter lifespans for women.

**Table 2:** Hazard ratios (HR) of first onset of age-related diseases from the main ICD-10 categories by age of death and sex

**ICD-10 category**	**HR (95% CI) for age of death**	**HR (95% CI) for sex (women)**	**HR (95% CI) for age of death: sex (women)**
Any disease	0.885 (0.884–0.885)	0.992 (0.986–0.998)	0.998 (0.997–0.998)
Neoplasms	0.848 (0.847–0.849)	0.719 (0.713–0.725)	0.975 (0.974–0.976)
Endocrine, nutritional, and metabolic diseases	0.894 (0.893–0.894)	1.059 (1.051–1.067)	1.011 (1.010–1.011)
Diseases of the nervous system	0.856 (0.854–0.857)	1.343 (1.325–1.361)	0.992 (0.990–0.993)
Diseases of the circulatory system	0.899 (0.899–0.900)	1.043 (1.036–1.049)	1.006 (1.005–1.007)
Diseases of the respiratory system	0.894 (0.893–0.895)	0.584 (0.578–0.590)	0.996 (0.994–0.997)
Diseases of the digestive system	0.890 (0.889–0.890)	0.906 (0.899–0.912)	0.998 (0.997–0.999)
Diseases of the musculoskeletal system and connective tissue	0.898 (0.897–0.898)	1.638 (1.626–1.651)	0.996 (0.996–0.997)
Diseases of the genitourinary system	0.852 (0.850–0.853)	0.922 (0.910–0.933)	1.005 (1.004–1.007)

The specific diseases belonging to each category can be found in Supplementary Table 1

*HR* Hazard ratio, *CI* Confidence interval

To account for multisystem involvement, we then analyzed multiple systems together. This allowed us to define whether this delay was observed only for independent systems, indicating system-specific deaccelerated aging with increasing lifespan, or was also observed with multisystem involvement, indicating globally deaccelerated aging. We computed survival curves to display the time to onset of a disease from the n^th^ ICD-10 category, from the second to the eighth category. We found that the survival curves followed the same pattern as when using the systems independently (Additional files 4 and 5), demonstrating a globally deaccelerated pace of aging in individuals with longer lifespans. Specifically, the HR for multisystem involvement gradually decreased as the number of systems affected increased (HR for 2 systems affected = 0.86, HR for 8 systems affected = 0.69). Interestingly, women had HR over 1 for a low number of systems affected (2 and 3 systems) and HR lower than 1 for the involvement of 5, 6, 7, and 8 systems, indicating higher hazard rates in women for developing diseases in a few systems, but greater protection from high multisystem involvement. Kaplan-Meier curves for 8 systems affected are not represented due to the globally reduced number of individuals with this condition, which was even lower in short and extremely long lifespans.

### Avoiding diseases

**Fig. 2:**
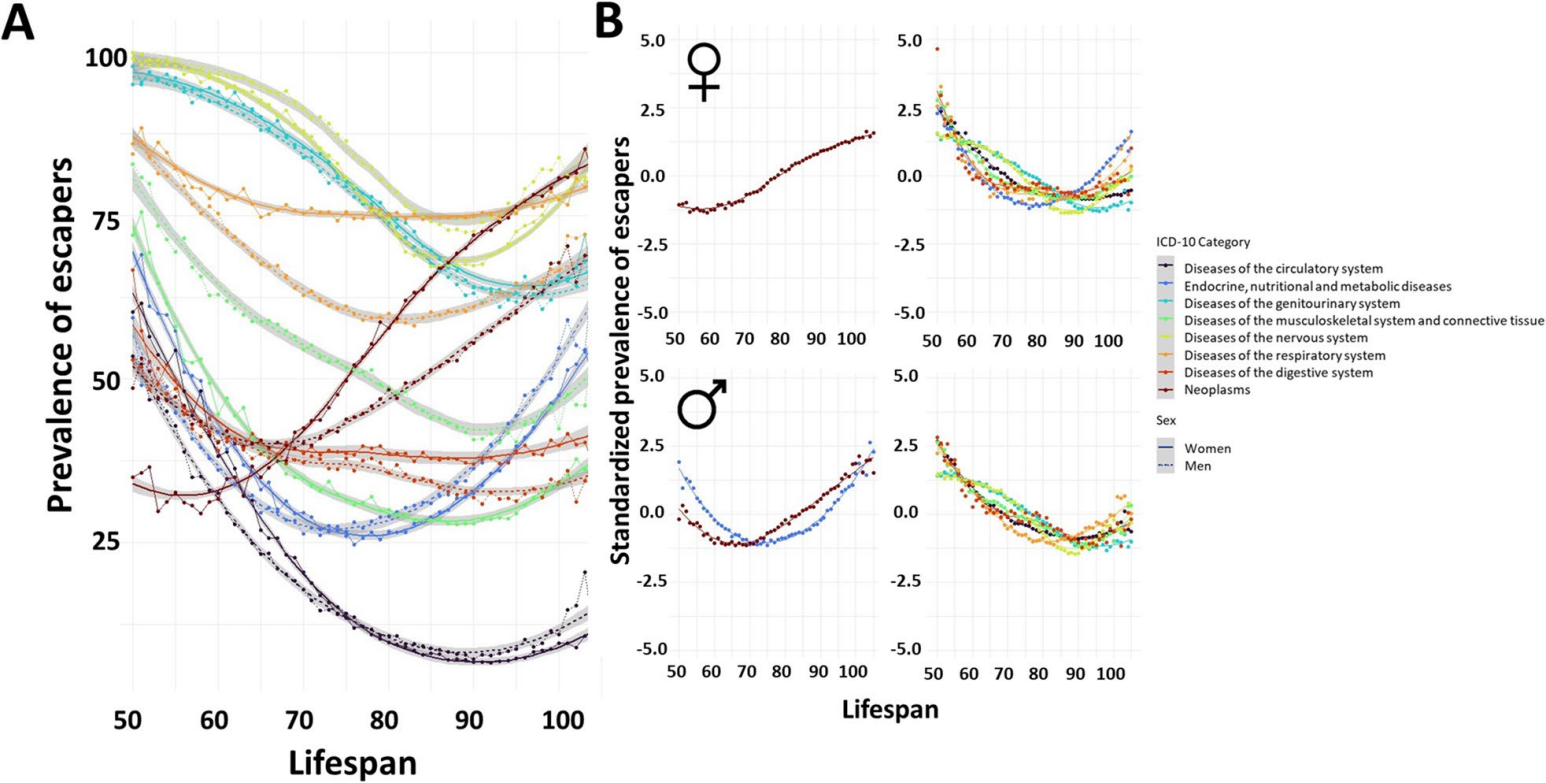
Evolution of the prevalence of escapers (individuals that died without a specific disease) according to the lifespan of the individuals. A higher prevalence of escapers indicates lower chance of developing the disease. **A** Overview of all the systems for both sexes. Dashed lines correspond to women, and solid lines correspond to men. **B** Systems grouped into similar evolution clusters using k-means clustering specific to each sex. The specific diseases belonging to each category can be found in Additional file 2

When studying the prevalence of escapers (participants who died without any disease), we observed a common pattern in all ICD-10 categories studied, showing a valley of escapers at different ages depending on the disease, in most cases between 70 and 90 years of lifespan (Fig. 2). The prevalence of escapers at the longest lifespans (105+) was similar to the prevalence in much shorter lifespans (< 75). When clustering the different evolutions of the prevalence of escapers for each system, two clusters appeared in both women and men. These clusters displayed similar trajectories and mainly differed in the lifespan at which the valley of escapers occurred, with an earlier valley in neoplasms in women and in neoplasms and endocrine, nutritional, and metabolic diseases in men.

Specifically, neoplasms displayed different curves in women than in men, both in shape and in levels of the prevalence of escapers. Endocrine, nutritional, and metabolic diseases displayed similar curves in women and men; however, the curve in women was delayed 4 years. Nervous system diseases had the same pattern in men and women, but the prevalence of escapers was lower in women. Escapers from diseases of the circulatory system showed similar patterns in both women and men. Regarding diseases of the respiratory system, the prevalence of escapers in women was higher than in men and was mainly stable across the different lifespans. Diseases of the digestive system displayed similar inverse exponential curves in men and women: the prevalence of escapers decreased more slowly for each year of lifespan increased. The prevalence of escapers from diseases of the musculoskeletal system and connective tissue was widely lower in women than in men. Finally, the prevalence of escapers from diseases of the genitourinary system displayed almost identical curves in both sexes. The specific prevalence of escapers for each main category and subcategory stratified by the decade of death can be found in Additional file 6.

**Fig. 3:**
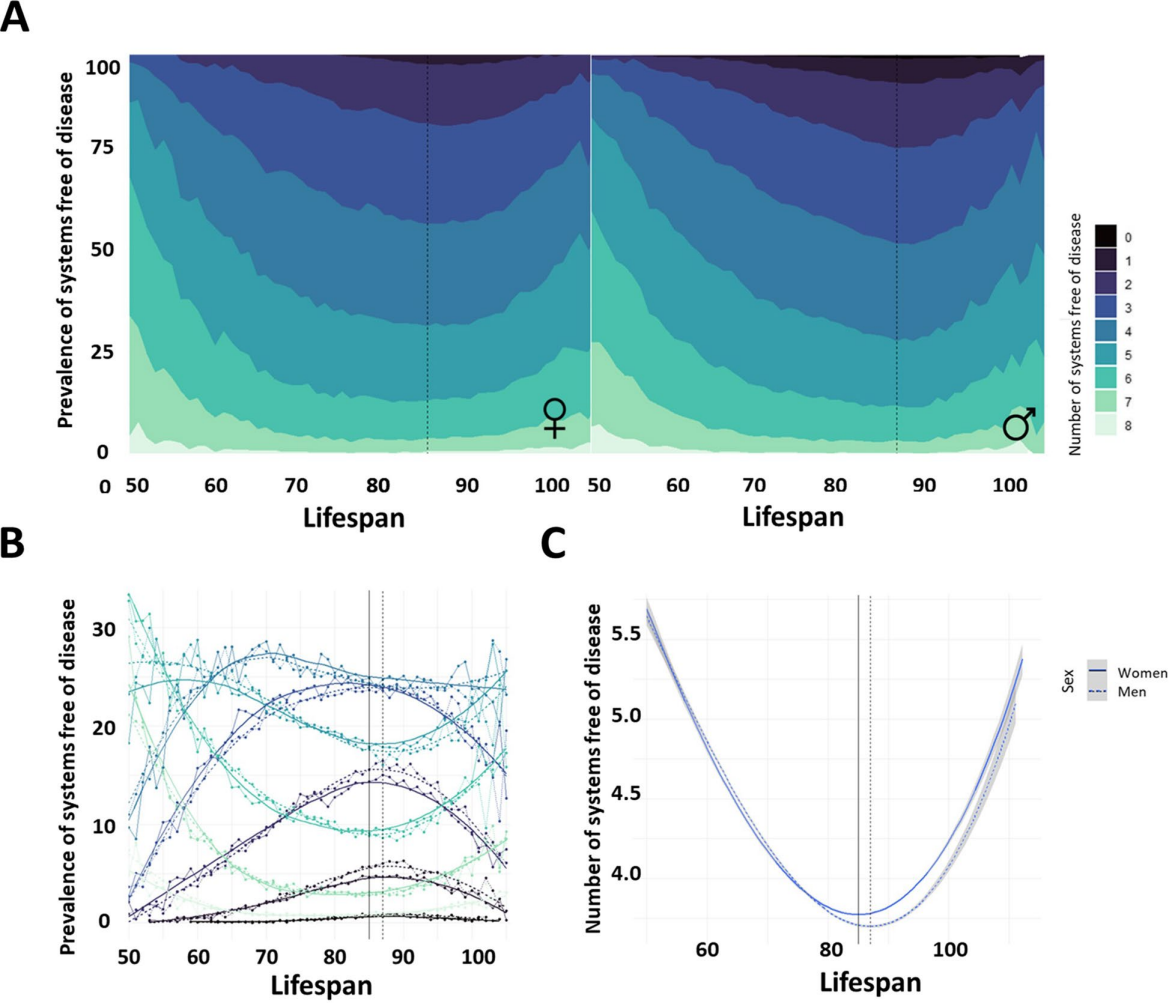
Evolution of multisystem involvement according to the lifespan of the individuals. **A** Cumulative prevalence of individuals with decreasing number of systems free of disease at death (from 8 to 0) according to the lifespan of the individuals, specifically for women and men. A higher cumulative prevalence indicates a globally higher number of systems free of disease at death. **C** Comparison by sex of the prevalence of individuals with each number of systems free of disease according to their lifespan. Dashed lines correspond to women, and solid lines correspond to men. **D** Evolution of the number of systems free of disease for both sexes. Dashed lines correspond to women, and solid lines correspond to men

If multiple systems were analyzed together by studying the number of systems free of disease, similar results were observed: the number of systems free of disease decreased until a certain age of death and then increased. Specifically, the lifespan with the minimum number of systems free of disease was 87 years for women and 88 years for men. These results were consistent with those obtained when analyzing the prevalence according to the main ICD-10 category and by subcategory (Fig. 3, Table 3).

**Table 3:** Prevalence of escapers of multisystem involvement according to the number of systems affected

**Death decade**	**All**		**50–59**		**60–69**		**70–79**		**80–89**		**90–99**		**100–105+**	
	**Women (*N* = 243,127)**	**Men (*N* = 238,931)**	**Women (*N* = 5224)**	**Men (*N* = 10,328)**	**Women (*N* = 13,648)**	**Men (*N* = 30,224)**	**Women (*N* = 35,106)**	**Men (*N* = 60,357)**	**Women (*N* = 103,706)**	**Men (*N* = 97,017)**	**Women (*N* = 80,457)**	**Men (*N* = 39,712)**	**Women (*N* = 4986)**	**Men (*N* = 1293)**
**Systems free of disease (continuous)**														
	3.97 [3.96;3.98]	3.98 [3.97;3.99]	5.15 [5.11;5.19]	5.16 [5.14;5.19]	4.41 [4.38;4.43]	4.46 [4.45;4.48]	3.99 [3.98;4.01]	4.01 [4.00;4.02]	3.83 [3.82;3.84]	3.75 [3.74;3.76]	3.96 [3.95;3.97]	3.82 [3.80;3.83]	4.44 [4.40;4.48]	4.36 [4.28;4.45]
**Systems free of disease (categorical)**														
0	0.48% [0.45%;0.51%]	0.50% [0.47%;0.53%]	0.02% [0.00%;0.11%]	0.00% [0.00%;0.04%]	0.10% [0.05%;0.16%]	0.03% [0.01%;0.06%]	0.25% [0.20%;0.31%]	0.28% [0.24%;0.32%]	0.61% [0.57%;0.66%]	0.73% [0.68%;0.79%]	0.51% [0.46%;0.56%]	0.76% [0.68%;0.85%]	0.24% [0.12%;0.42%]	0.31% [0.08%;0.79%]
1	3.81% [3.73%;3.88%]	4.02% [3.94%;4.10%]	0.29% [0.16%;0.47%]	0.20% [0.13%;0.31%]	1.20% [1.03%;1.40%]	1.02% [0.91%;1.14%]	2.89% [2.72%;3.07%]	3.13% [2.99%;3.27%]	4.47% [4.34%;4.59%]	5.43% [5.29%;5.57%]	4.11% [3.97%;4.25%]	5.24% [5.02%;5.46%]	2.51% [2.09%;2.98%]	3.09% [2.22%;4.19%]
2	12.5% [12.4%;12.7%]	12.5% [12.4%;12.7%]	3.37% [2.90%;3.89%]	2.23% [1.95%;2.53%]	6.93% [6.51%;7.37%]	6.48% [6.21%;6.77%]	11.6% [11.2%;11.9%]	11.8% [11.6%;12.1%]	14.1% [13.9%;14.3%]	15.2% [15.0%;15.4%]	12.9% [12.6%;13.1%]	14.5% [14.2%;14.9%]	7.92% [7.19%;8.71%]	8.20% [6.76%;9.83%]
3	22.5% [22.4%;22.7%]	21.8% [21.6%;22.0%]	9.19% [8.42%;10.0%]	9.21% [8.66%;9.78%]	18.8% [18.2%;19.5%]	17.7% [17.3%;18.1%]	23.3% [22.8%;23.7%]	22.3% [22.0%;22.7%]	23.9% [23.6%;24.1%]	23.6% [23.3%;23.9%]	22.4% [22.1%;22.7%]	23.0% [22.6%;23.4%]	17.1% [16.1%;18.2%]	17.9% [15.9%;20.1%]
4	25.0% [24.8%;25.2%]	24.8% [24.6%;25.0%]	19.2% [18.1%;20.3%]	20.0% [19.2%;20.7%]	26.4% [25.6%;27.1%]	26.5% [26.0%;27.0%]	26.6% [26.1%;27.0%]	26.0% [25.6%;26.3%]	24.9% [24.6%;25.2%]	24.5% [24.2%;24.7%]	24.7% [24.4%;25.0%]	23.9% [23.5%;24.4%]	23.5% [22.4%;24.7%]	24.1% [21.7%;26.5%]
5	19.5% [19.3%;19.7%]	19.9% [19.7%;20.0%]	25.2% [24.0%;26.4%]	27.1% [26.2%;27.9%]	24.2% [23.4%;24.9%]	25.0% [24.5%;25.5%]	20.4% [20.0%;20.9%]	20.8% [20.5%;21.1%]	18.4% [18.2%;18.7%]	17.7% [17.5%;17.9%]	19.1% [18.8%;19.4%]	17.9% [17.5%;18.3%]	23.4% [22.2%;24.6%]	23.0% [20.7%;25.4%]
6	11.0% [10.9%;11.1%]	11.4% [11.3%;11.5%]	25.1% [23.9%;26.3%]	24.0% [23.2%;24.8%]	15.2% [14.6%;15.8%]	16.0% [15.6%;16.4%]	10.9% [10.6%;11.2%]	11.3% [11.1%;11.6%]	9.52% [9.34%;9.70%]	9.16% [8.98%;9.34%]	11.0% [10.7%;11.2%]	10.0% [9.72%;10.3%]	16.0% [15.0%;17.0%]	14.2% [12.3%;16.2%]
7	4.02% [3.94%;4.10%]	4.11% [4.03%;4.19%]	14.5% [13.6%;15.5%]	13.6% [13.0%;14.3%]	6.14% [5.74%;6.56%]	6.02% [5.75%;6.29%]	3.37% [3.19%;3.57%]	3.58% [3.44%;3.74%]	3.20% [3.10%;3.31%]	2.97% [2.86%;3.07%]	4.12% [3.99%;4.26%]	3.66% [3.48%;3.85%]	7.22% [6.52%;7.97%]	7.12% [5.77%;8.66%]
8	1.11% [1.07%;1.15%]	0.98% [0.94%;1.02%]	3.14% [2.68%;3.65%]	3.70% [3.34%;4.08%]	1.15% [0.98%;1.34%]	1.25% [1.13%;1.38%]	0.79% [0.70%;0.89%]	0.76% [0.69%;0.83%]	0.95% [0.89%;1.01%]	0.76% [0.70%;0.81%]	1.26% [1.18%;1.34%]	0.92% [0.83%;1.02%]	2.09% [1.71%;2.52%]	2.17% [1.44%;3.11%]

### Health span and percentage of life free of disease

**Fig. 4:**
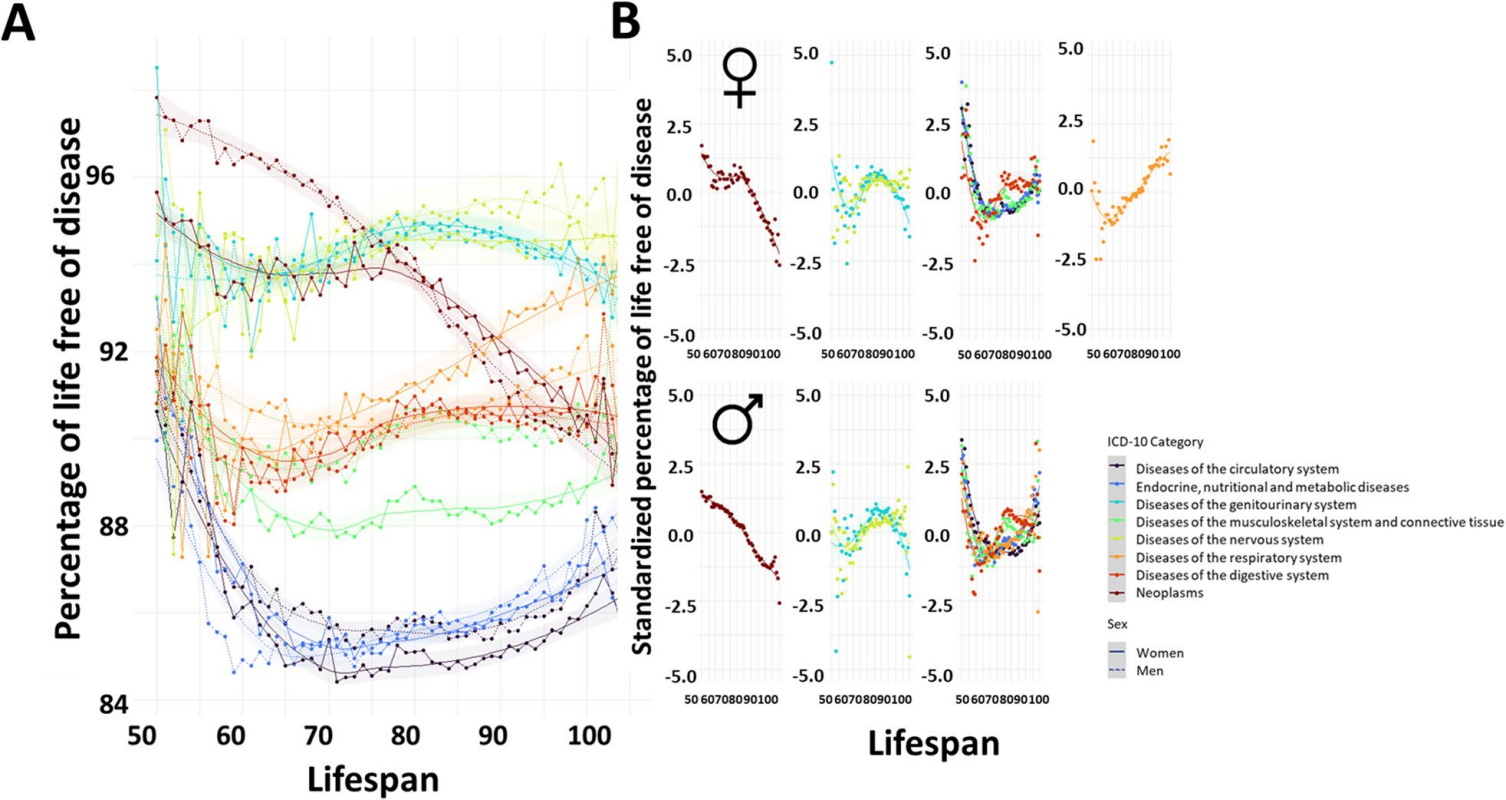
Evolution of the percentage of life free of disease according to the lifespan of the individuals. Higher percentages of life free of disease indicate longer health spans. **A** Overview of all the systems for both sexes. Dashed lines correspond to women, and solid lines correspond to men. **B** Systems grouped into similar evolution clusters using k-means clustering specific to each sex. The specific diseases belonging to each category can be found in Additional file 2

We then focused on non-escaper individuals. We analyzed the percentage of life free of disease (time from birth to the onset of the disease relative to years of life) of non-escapers in order to determine the contribution of these diseases to the disability of elderly individuals and to establish the focus of interventions for extending lifespan and health span. Here, a diversity of trajectories was observed. We focused on clusters of ICD-10 categories with trajectories of similar behavior regarding the relationship between years of disease and lifespan. We found 4 main clusters in women and 3 in men (Fig. 4).

Cluster 1 in both women and men included neoplasms, with a percentage of life free of disease that displayed a linear reduction as lifespan increased. In women, the decrease in the percentage of years free of neoplasms was more pronounced in those individuals who died after their 80 s. These results indicate that the most long-lived individuals that suffered neoplasms lived more years following diagnosis.

Diseases of the nervous system and diseases of the genitourinary system (cluster 2) remained mainly stable across all lifespans. A slight valley and peak could be observed at lifespan 65 and 80, respectively, except for diseases of the nervous system in women, in which the curve slightly increased with lifespan.

Diseases of the digestive system, diseases of the musculoskeletal system and connective tissue, diseases of the circulatory system, and endocrine, nutritional, and metabolic diseases were included in cluster 3 in both women and men. Additionally, cluster 3 in men also included diseases of the respiratory system. The percentage of life free of these diseases decreased up to the lifespan of 65. Afterwards, it increased slightly until lifespans of 105+ following slightly different patterns, suggesting partially decelerated aging of these systems in individuals that lived more than 65 years.

Finally, cluster 4 contained diseases of the respiratory system in women. The analysis showed that the percentage of life free of disease decreased between 50 and 70 years of lifespan and increased thereafter, indicating a delay in the onset of diseases in this system in lifespans over 70 years.

### MFA with pathologies at death and percentage of life free of disease

**Fig. 5:**
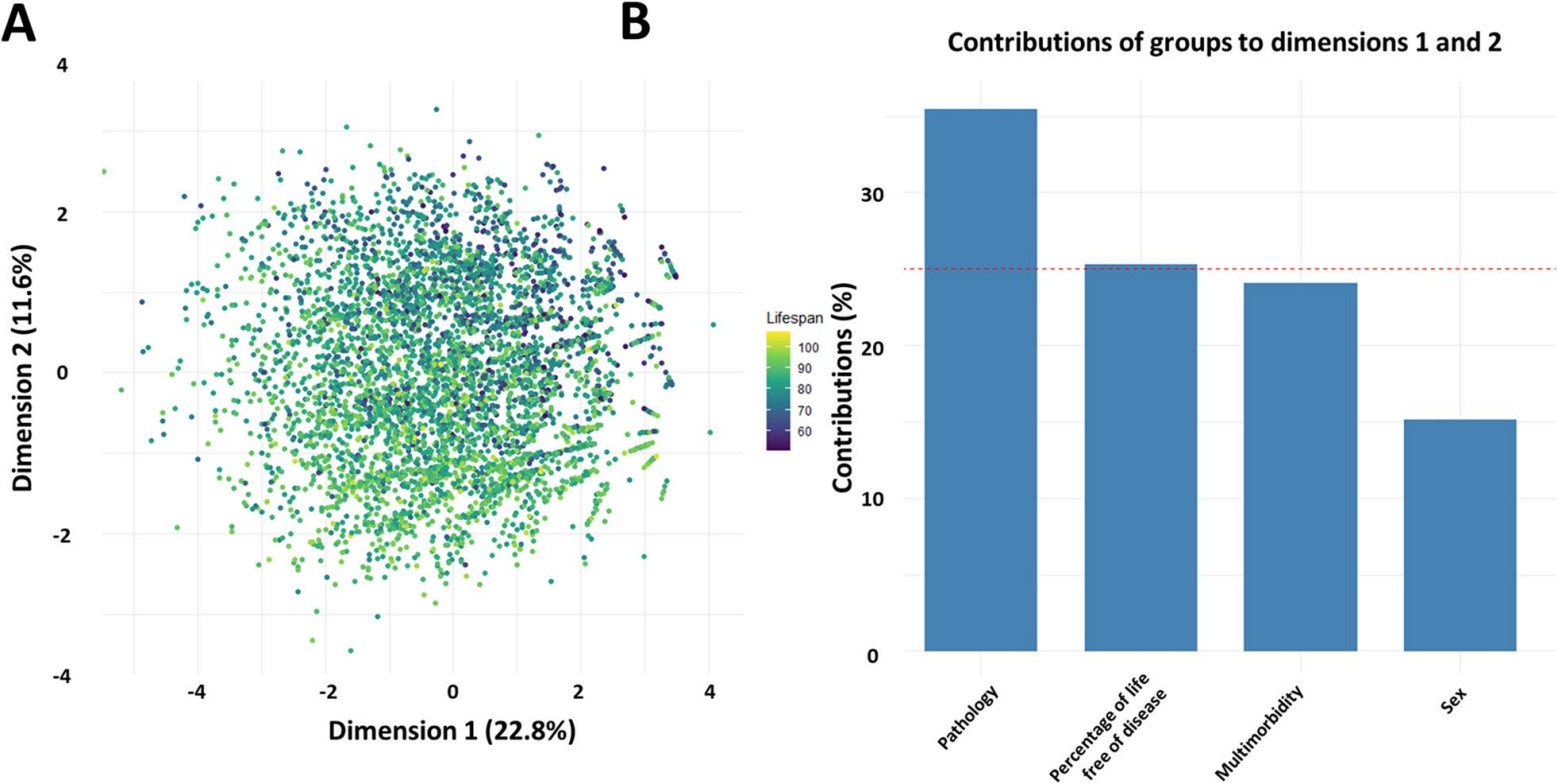
Multiple factor analysis on sex, number of systems free of disease, involvement in a system, and the percentage of life free of disease in the different systems. **A** Score plots from a random subset of 4821 individuals colored according to their lifespan. Closer points correspond to more similar individuals. **B** contribution of the groups of variables to the first two dimensions. Dashed red line: expected value considering uniform contributions

Based on the differences observed, we then assessed whether these variables were able to explain part of the variability in lifespan. We performed an MFA including involvement of the different systems (yes/no), the percentage of life free of disease, and the number of systems free of disease. When representing a score plot on a random subset of 1% of the samples on the first two dimensions of the MFA, individuals were distributed according to their lifespan (Fig. 5A). The involvement of the system and the percentage of life free of disease had great contributions to these two dimensions (Fig. 5B).

## Discussion

In this study, we analyzed the relationship between lifespan and age of onset, avoidance, and percentage of life free of diseases to define their contribution to lifespan. We describe a gradual delay in the age of onset of all diseases as lifespan increases, both single and multisystem, indicating that globally, individuals with greater lifespan have a specific resistance to developing diseases and can delay their onset. The delay was observed in both lethal (e.g., neoplasms) and non-lethal diseases (e.g., diseases of the musculoskeletal system and connective tissue) indicating a globally slower pace of aging in longer-lived individuals. These results agree with those reported in extremely long-lived individuals (from 100 to 119 years old) that demonstrated that those individuals present a later onset of frailty and diseases such as cancer, cardiovascular disease, dementia, and stroke, as well as cognitive and functional decline [21], and reinforce the effectiveness of disease prevention for increasing lifespan rather than curing diseases. In the same line, long-lived families can delay or avoid age-related diseases to a greater extent than shorter-lived families [28]. Furthermore, our results show that the HR gradually decreases as the number of systems affected increases, indicating that this resistance is enhanced for multisystem involvement.

When considering the prevalence of escapers, we observed that this prevalence was lower in lifespans around life expectancy (in most diseases, between lifespans of 70 and 90 years) depending on the system and that individuals with the longest lifespans had a similar prevalence of escapers than those with the shortest ones. These results could be explained by a higher severity of specific diseases in individuals with shorter lifespans, which may prevent the onset of other diseases before death [29], in contrast with a higher protection against developing diseases in longer-lived individuals, as suggested by other authors [30, 31]. We also observed how globally, the number of systems free of disease decreased until the ages of death 87–88 and started increasing thereafter, indicating that up to these ages (87–88) individuals die with more systems affected as their lifespan increases whereas after these ages, individuals progressively die with less systems affected as their lifespan increases. Similarly, a previous study performed with the SIDIAP database described how before 85 years of age frailty is mainly associated with the number of concurrent diseases, and that from 85 years onwards frailty is associated with disability and other signs and symptoms [32]. These results support the existence of a major survival hurdle at 87–88 years old, defining two main groups of individuals in terms of their lifespan: those who die before and those who die after. Before these ages, our results confirm the age-dependent loss of homeostasis in a system-specific fashion. Individuals that die after 87–88 years of age may have greater protection against suffering age-related diseases, which may be the result of optimized homeostatic protective mechanisms. Until now, there has been a consensus that considers centenarians as chronological age-based models of successful aging [30], together with other individual-centered models such as the one proposed by Rowe and Kahn [33]. However, based on our data, we propose that centenarians are the most extreme case of successful aging, and individuals with lifespans over 87–88 years are also relevant for the study of extreme lifespan. Consequently, a better characterization of the individual pace of aging in these subjects should provide valuable information on how to delay the onset of chronic diseases and increase health span and lifespan in the whole population. In this sense, a deep molecular characterization of the biology of aging in human tissues is crucial to better understand the meaning of human longevity and should serve as a reference to identify longevous and non-longevous individuals among middle-aged adults to better define individualized health strategies.

Focusing on sex-specific patterns, we found remarkably low HR and higher prevalence of escapers in women for diseases of the respiratory system and neoplasms at lifespans around life expectancy, as well as remarkably high HR and lower prevalence of escapers for diseases of the musculoskeletal system and connective tissue and for diseases of the nervous system. Neoplasms and diseases of the respiratory system (excluding infectious diseases) are the second and fourth leading causes of death in Spain [34]. Advanced pulmonary age has been described as the strongest predictor of mortality in the UK Biobank study [20], as well as an important mortality risk factor in epidemiological studies [35]. In contrast, diseases of the nervous system are the third cause of death, and diseases of the musculoskeletal system and connective tissue are the last cause of mortality among the studied diseases [34]. Both diseases of the nervous system and diseases of the musculoskeletal system have significantly higher survival times [36] and are among the main causes of frailty [25, 37]. These results could be explained by the differences in metabolic regulation in women and men during the aging process. For instance, previous results from our group described a higher involvement of gut-derived aromatic amino acids in women, which are strongly related to cognitive function and could lead to a higher risk of developing age-related nervous system diseases, as described in this work [38]. Furthermore, our study reveals that multisystem involvement is lower in women than in men at longer lifespans, contrarily to most of the current literature, which describes higher multimorbidity in women, specifically at older ages [32, 39]. One possible explanation is that we evaluated the number of systems affected instead of global multimorbidity. These differences imply that men and women achieve the same lifespan through different mechanisms. In women, the capacity to delay the onset of more lethal diseases and multisystem involvement could be greater, while men may be able to survive them for longer times. This could partly explain why life expectancy in women is greater, as mortality is closely associated with disease-related frailty [32]. In line with this hypothesis, we previously described sex-specific metabolic regulation during aging [38], that could provide women a better adaptation to the aging process and a higher resistance to multisystem diseases. Notably, the hazard rate for neoplasms in women at younger ages of death was higher than in men, in line with the increase in the incidence of prostate cancer in men after 65 years of age compared with the decrease in breast cancer after this age in women [40].

When analyzing the percentage of life free of disease in non-escapers, we found important differences related to the system that could explain human lifespan. Our results suggest that in people who suffer neoplasms, lifespan is strongly related to survival. In contrast, in the case of diseases of the respiratory system (men and women), and endocrine, nutritional, and metabolic diseases (men), lifespan is related to the ability of individuals to delay these diseases. In contrast with other chronic age-related diseases such as diabetes mellitus, Alzheimer’s disease, hypertension, or chronic obstructive pulmonary disease, to name some of the most prevalent diseases from other systems, neoplasms present an earlier onset, higher short or mid-term mortality and, although considered as a chronic disease, could be a one-time event [41]. These results highlight the importance of understanding the underlying mechanisms leading to each pathology to better comprehend their etiology.

Integrating the variables related to escaping the disease and the percentage of life spent free of disease at the time of death in an MFA, we found that these variables alone can contribute to explaining variability in individual lifespan.

This study has some limitations: (i) It was based on real-world data and specifically EHR, so it may include inconsistencies derived from data entry during clinical practice. However, these are expected to be small in number and minimized by the size and representativeness of the study population. (ii) Data before 2005 were collected in paper records, and some of the data, such as completeness of the registers and dates of diagnosis, might be less accurate. However, the fact that the results are sex- and system-dependent imparts a trustworthiness of there not being a generalized inconsistency. (iii) The analysis did not include confounding variables. This was because the main confounders that may be used can be interpreted as the cause of accelerated or decelerated aging rather than purely confounding variables. (iv) The severity of the disease was not taken into account. The wide scope of the present study rendered it impossible to specify each disease. Further system-specific studies discriminating between specific diseases must be performed. (v) The study population exhibited bias in terms of race and ethnicity, as 80% of the individuals were from Spain, 5.1% from other European regions, 6.2% from America, 4.9% from Africa, 3.1% from Asia, and less than 0.1% from Oceania. Consequently, this fact may limit the generalizability of the results to all regions worldwide.

## Conclusions

**Fig. 6:**
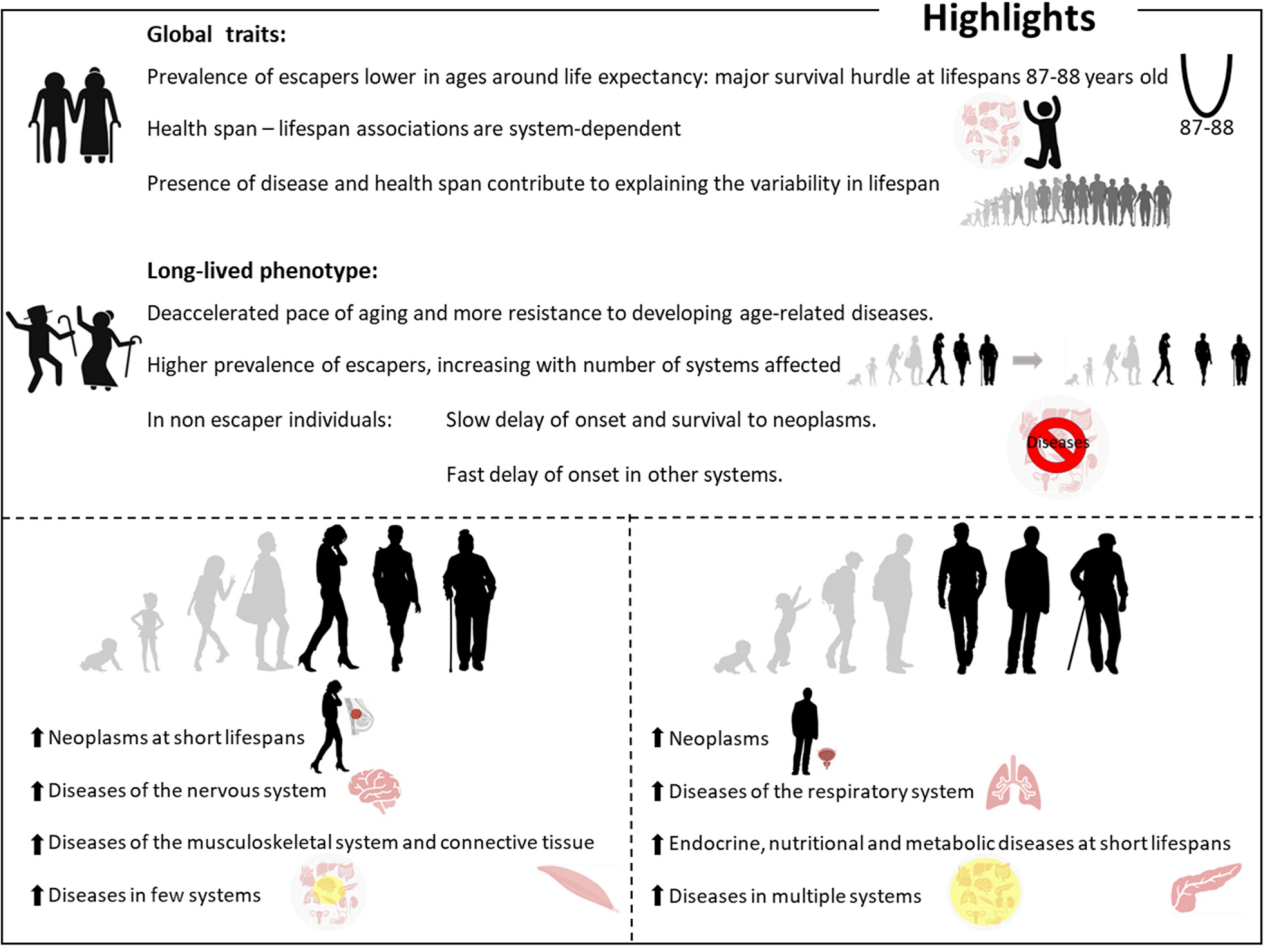
Summary of the main results of the study. Images partially created with BioRender.com

The key findings of this study are specified in Fig. 6 and can be summarized as (i) the age of onset of disease, both single and multisystem, is gradually delayed as lifespan increases, (ii) the prevalence of escapers is lower in ages around life expectancy, (iii) the number of systems free of diseases decreases until ages of death 87–88 years old and increases onwards, (iv) long-lived women are less prone to multisystem involvement, (v) health span-lifespan associations are system-dependent, (vi) disease and percentage of life free of disease at death contribute to explaining the variability of lifespan, and (vii) there are system-specific differences between women and men.

Health interventions focused on delaying aging and age-related diseases should be the most effective in increasing not only lifespan but also health span. The findings of this research highlight the relevance of EHR in studying the aging process and open up new possibilities in age-related disease prevention that should assist primary care professionals in devising individualized care and treatment plans.

### Supplementary Information

**Additional file 1.** Flow chart of the study population. The number of individuals that do not meet each inclusion criterion are reported, together with the reason of exclusion.

**Additional file 2.** List of ICD-10 codes analysed.

**Additional file 3.** Data regarding numbers at risk for the survival data.

**Additional file 4.** Kaplan-Meier curves for multisystem involvement in different biological systems according to age of death and stratified by sex, accounting for 2 to 7 systems affected. The proportion of individuals free of disease (y axis) at each age (x axis) is represented stratifying by lifespan of individuals and sex. Each panel includes the curves from both sexes corresponding to the same number of systems affected. Curves that decay earlier indicate higher risk of an early onset of diseases in multiple systems. Age of onset is considered as the age of diagnosis of the first disease in the n^th^ system.

**Additional file 5.** Hazard ratios (HR) for multisystem involvement in different biological systems from the main ICD-10 categories by age of death and sex.

**Additional file 6.** Prevalence of escapers according to ICD-10 categories, by sex and decade of death.

## Data Availability

In accordance with current European and national law, the data used in our study are only available for the researchers participating in our study. Thus, we are not allowed to distribute or make publicly available the data to other parties. Researchers from public institutions can request data from SIDIAP if they comply with certain requirements. Further information is available online (https://www.sidiap.org/index.php/menu-solicitudesen/application-proccedure).

## Abbreviations

EHR: Electronic health records
HR: Hazard ratios
ICD-10: International Classification of Diseases, Tenth Revision
LOESS: Locally estimated scatterplot smoothing
MFA: Multiple factor analysis
P4 medicine: Predictive, preventive, personalized, and participatory medicine
SIDIAP: Information System for the Development of Research in Primary Care
