# Supplementary figures and images for: Human lifespan and sex-specific patterns of resilience to disease: a retrospective population-wide cohort study
Source: BMC Med. 2024 Jan 8;22:17. doi: 10.1186/s12916-023-03206-w (PMC10773063; doi:10.1186/s12916-023-03206-w)

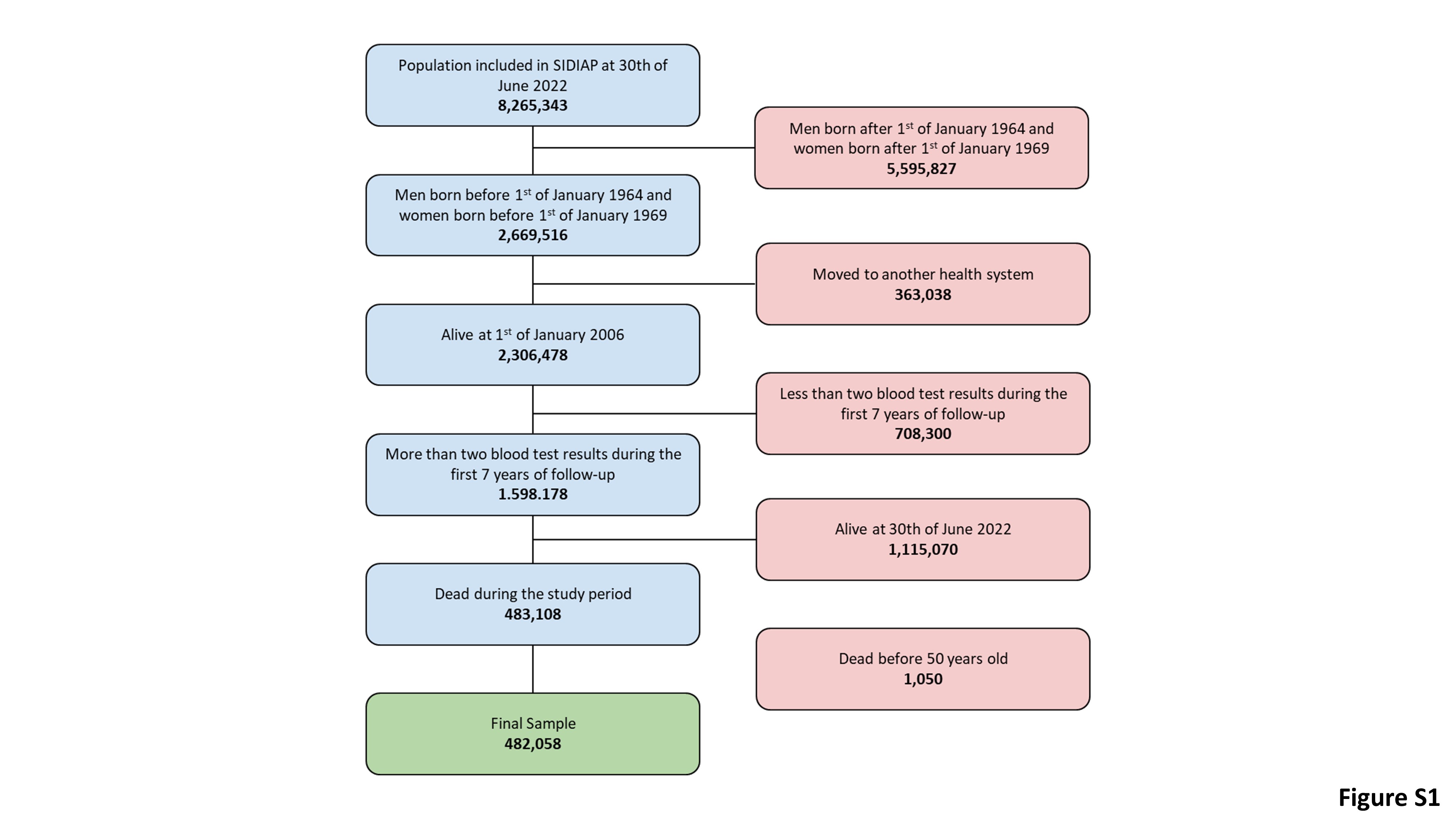

Supplement: Supplementary file 1 — Additional file 1. Flow chart of the study population. The number of individuals that do not meet each inclusion criterion are reported, together with the reason of exclusion. [file 12916_2023_3206_MOESM1_ESM.jpg]

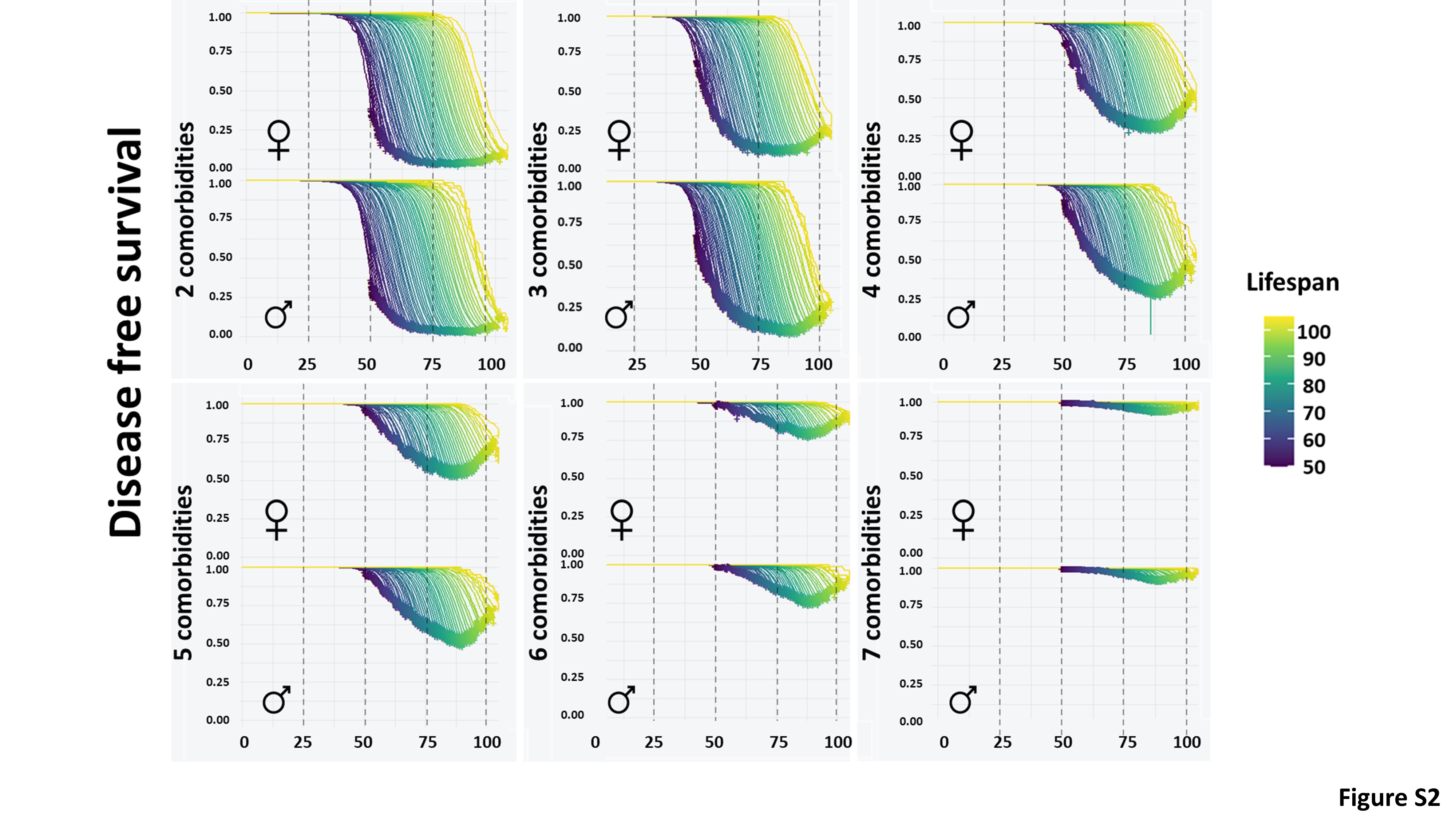

Supplement: Supplementary file 4 — Additional file 4. Kaplan-Meier curves for multisystem involvement in different biological systems according to age of death and stratified by sex, accounting for 2 to 7 systems affected. The proportion of individuals free of disease (y axis) at each age (x axis) is represented stratifying by lifespan of individuals and sex. Each panel includes the curves from both sexes corresponding to the same number of systems affected. Curves that decay earlier indicate higher risk of an early onset of diseases in multiple systems. Age of onset is considered as the age of diagnosis of the first disease in the nth system. [file 12916_2023_3206_MOESM4_ESM.jpg]
