# Supplementary material for: Human lifespan and sex-specific patterns of resilience to disease: a retrospective population-wide cohort study
Source: BMC Med. 2024 Jan 8;22:17. doi: 10.1186/s12916-023-03206-w (PMC10773063; doi:10.1186/s12916-023-03206-w)
Supplement: Supplementary file 2 — Additional file 2. List of ICD-10 codes analysed. [file 12916_2023_3206_MOESM2_ESM.docx]

Table S1. List of ICD-10 codes analysed

| ICD-10 category | ICD-10 chapter | Main ICD-10 category | ICD-10 Group | Disease |
| --- | --- | --- | --- | --- |
| C00 | C | Neoplasms | Malignant neoplasms | Malignant neoplasm of lip |
| C01 | C | Neoplasms | Malignant neoplasms | Malignant neoplasm of base of tongue |
| C02 | C | Neoplasms | Malignant neoplasms | Malignant neoplasm of other and unspecified parts of tongue |
| C03 | C | Neoplasms | Malignant neoplasms | Malignant neoplasm of gum |
| C04 | C | Neoplasms | Malignant neoplasms | Malignant neoplasm of floor of mouth |
| C05 | C | Neoplasms | Malignant neoplasms | Malignant neoplasm of palate |
| C06 | C | Neoplasms | Malignant neoplasms | Malignant neoplasm of other and unspecified parts of mouth |
| C07 | C | Neoplasms | Malignant neoplasms | Malignant neoplasm of parotid gland |
| C08 | C | Neoplasms | Malignant neoplasms | Malignant neoplasm of other and unspecified major salivary glands |
| C09 | C | Neoplasms | Malignant neoplasms | Malignant neoplasm of tonsil |
| C10 | C | Neoplasms | Malignant neoplasms | Malignant neoplasm of oropharynx |
| C11 | C | Neoplasms | Malignant neoplasms | Malignant neoplasm of nasopharynx |
| C12 | C | Neoplasms | Malignant neoplasms | Malignant neoplasm of piriform sinus |
| C13 | C | Neoplasms | Malignant neoplasms | Malignant neoplasm of hypopharynx |
| C14 | C | Neoplasms | Malignant neoplasms | Malignant neoplasm of other and ill-defined sites in the lip, oral cavity and pharynx |
| C15 | C | Neoplasms | Malignant neoplasms | Malignant neoplasm of oesophagus |
| C16 | C | Neoplasms | Malignant neoplasms | Malignant neoplasm of stomach |
| C17 | C | Neoplasms | Malignant neoplasms | Malignant neoplasm of small intestine |
| C18 | C | Neoplasms | Malignant neoplasms | Malignant neoplasm of colon |
| C19 | C | Neoplasms | Malignant neoplasms | Malignant neoplasm of rectosigmoid junction |
| C20 | C | Neoplasms | Malignant neoplasms | Malignant neoplasm of rectum |
| C21 | C | Neoplasms | Malignant neoplasms | Malignant neoplasm of anus and anal canal |
| C22 | C | Neoplasms | Malignant neoplasms | Malignant neoplasm of liver and intrahepatic bile ducts |
| C23 | C | Neoplasms | Malignant neoplasms | Malignant neoplasm of gallbladder |
| C24 | C | Neoplasms | Malignant neoplasms | Malignant neoplasm of other and unspecified parts of biliary tract |
| C25 | C | Neoplasms | Malignant neoplasms | Malignant neoplasm of pancreas |
| C26 | C | Neoplasms | Malignant neoplasms | Malignant neoplasm of other and ill-defined digestive organs |
| C30 | C | Neoplasms | Malignant neoplasms | Malignant neoplasm of nasal cavity and middle ear |
| C31 | C | Neoplasms | Malignant neoplasms | Malignant neoplasm of accessory sinuses |
| C32 | C | Neoplasms | Malignant neoplasms | Malignant neoplasm of larynx |
| C33 | C | Neoplasms | Malignant neoplasms | Malignant neoplasm of trachea |
| C34 | C | Neoplasms | Malignant neoplasms | Malignant neoplasm of bronchus and lung |
| C37 | C | Neoplasms | Malignant neoplasms | Malignant neoplasm of thymus |
| C38 | C | Neoplasms | Malignant neoplasms | Malignant neoplasm of heart, mediastinum and pleura |
| C39 | C | Neoplasms | Malignant neoplasms | Malignant neoplasm of other and ill-defined sites in the respiratory system and intrathoracic organs |
| C40 | C | Neoplasms | Malignant neoplasms | Malignant neoplasm of bone and articular cartilage of limbs |
| C41 | C | Neoplasms | Malignant neoplasms | Malignant neoplasm of bone and articular cartilage of other and unspecified sites |
| C45 | C | Neoplasms | Malignant neoplasms | Mesothelioma |
| C46 | C | Neoplasms | Malignant neoplasms | Kaposi sarcoma |
| C47 | C | Neoplasms | Malignant neoplasms | Malignant neoplasm of peripheral nerves and autonomic nervous system |
| C48 | C | Neoplasms | Malignant neoplasms | Malignant neoplasm of retroperitoneum and peritoneum |
| C49 | C | Neoplasms | Malignant neoplasms | Malignant neoplasm of other connective and soft tissue |
| C4A | C | Neoplasms | Malignant neoplasms | Merkel cell carcinoma |
| C50 | C | Neoplasms | Malignant neoplasms | Malignant neoplasm of breast |
| C51 | C | Neoplasms | Malignant neoplasms | Malignant neoplasm of vulva |
| C52 | C | Neoplasms | Malignant neoplasms | Malignant neoplasm of vagina |
| C53 | C | Neoplasms | Malignant neoplasms | Malignant neoplasm of cervix uteri |
| C54 | C | Neoplasms | Malignant neoplasms | Malignant neoplasm of corpus uteri |
| C55 | C | Neoplasms | Malignant neoplasms | Malignant neoplasm of uterus, part unspecified |
| C56 | C | Neoplasms | Malignant neoplasms | Malignant neoplasm of ovary |
| C57 | C | Neoplasms | Malignant neoplasms | Malignant neoplasm of other and unspecified female genital organs |
| C58 | C | Neoplasms | Malignant neoplasms | Malignant neoplasm of placenta |
| C60 | C | Neoplasms | Malignant neoplasms | Malignant neoplasm of penis |
| C61 | C | Neoplasms | Malignant neoplasms | Malignant neoplasm of prostate |
| C62 | C | Neoplasms | Malignant neoplasms | Malignant neoplasm of testis |
| C63 | C | Neoplasms | Malignant neoplasms | Malignant neoplasm of other and unspecified male genital organs |
| C64 | C | Neoplasms | Malignant neoplasms | Malignant neoplasm of kidney, except renal pelvis |
| C65 | C | Neoplasms | Malignant neoplasms | Malignant neoplasm of renal pelvis |
| C66 | C | Neoplasms | Malignant neoplasms | Malignant neoplasm of ureter |
| C67 | C | Neoplasms | Malignant neoplasms | Malignant neoplasm of bladder |
| C68 | C | Neoplasms | Malignant neoplasms | Malignant neoplasm of other and unspecified urinary organs |
| C69 | C | Neoplasms | Malignant neoplasms | Malignant neoplasm of eye and adnexa |
| C70 | C | Neoplasms | Malignant neoplasms | Malignant neoplasm of meninges |
| C71 | C | Neoplasms | Malignant neoplasms | Malignant neoplasm of brain |
| C72 | C | Neoplasms | Malignant neoplasms | Malignant neoplasm of spinal cord, cranial nerves and other parts of central nervous system |
| C73 | C | Neoplasms | Malignant neoplasms | Malignant neoplasm of thyroid gland |
| C74 | C | Neoplasms | Malignant neoplasms | Malignant neoplasm of adrenal gland |
| C75 | C | Neoplasms | Malignant neoplasms | Malignant neoplasm of other endocrine glands and related structures |
| C76 | C | Neoplasms | Malignant neoplasms | Malignant neoplasm of other and ill-defined sites |
| C77 | C | Neoplasms | Malignant neoplasms | Secondary and unspecified malignant neoplasm of lymph nodes |
| C78 | C | Neoplasms | Malignant neoplasms | Secondary malignant neoplasm of respiratory and digestive organs |
| C79 | C | Neoplasms | Malignant neoplasms | Secondary malignant neoplasm of other and unspecified sites |
| C7A | C | Neoplasms | Malignant neoplasms | Malignant neuroendocrine tumors |
| C7B | C | Neoplasms | Malignant neoplasms | Secondary neuroendocrine tumors |
| C80 | C | Neoplasms | Malignant neoplasms | Malignant neoplasm, without specification of site |
| C81 | C | Neoplasms | Malignant neoplasms | Hodgkin lymphoma |
| C82 | C | Neoplasms | Malignant neoplasms | Follicular lymphoma |
| C83 | C | Neoplasms | Malignant neoplasms | Non-follicular lymphoma |
| C84 | C | Neoplasms | Malignant neoplasms | Mature T/NK-cell lymphomas |
| C85 | C | Neoplasms | Malignant neoplasms | Other and unspecified types of non-Hodgkin lymphoma |
| C86 | C | Neoplasms | Malignant neoplasms | Other specified types of T/NK-cell lymphoma |
| C88 | C | Neoplasms | Malignant neoplasms | Malignant immunoproliferative diseases |
| C90 | C | Neoplasms | Malignant neoplasms | Multiple myeloma and malignant plasma cell neoplasms |
| C91 | C | Neoplasms | Malignant neoplasms | Lymphoid leukaemia |
| C92 | C | Neoplasms | Malignant neoplasms | Myeloid leukaemia |
| C93 | C | Neoplasms | Malignant neoplasms | Monocytic leukaemia |
| C94 | C | Neoplasms | Malignant neoplasms | Other leukaemias of specified cell type |
| C95 | C | Neoplasms | Malignant neoplasms | Leukaemia of unspecified cell type |
| C96 | C | Neoplasms | Malignant neoplasms | Other and unspecified malignant neoplasms of lymphoid, haematopoietic and related tissue |
| D00 | D | Neoplasms | In situ neoplasms | Carcinoma in situ of oral cavity, oesophagus and stomach |
| D01 | D | Neoplasms | In situ neoplasms | Carcinoma in situ of other and unspecified digestive organs |
| D02 | D | Neoplasms | In situ neoplasms | Carcinoma in situ of middle ear and respiratory system |
| D03 | D | Neoplasms | In situ neoplasms | Melanoma in situ |
| D04 | D | Neoplasms | In situ neoplasms | Carcinoma in situ of skin |
| D05 | D | Neoplasms | In situ neoplasms | Carcinoma in situ of breast |
| D06 | D | Neoplasms | In situ neoplasms | Carcinoma in situ of cervix uteri |
| D07 | D | Neoplasms | In situ neoplasms | Carcinoma in situ of other and unspecified genital organs |
| D09 | D | Neoplasms | In situ neoplasms | Carcinoma in situ of other and unspecified sites |
| D10 | D | Neoplasms | Benign neoplasms | Benign neoplasm of mouth and pharynx |
| D11 | D | Neoplasms | Benign neoplasms | Benign neoplasm of major salivary glands |
| D12 | D | Neoplasms | Benign neoplasms | Benign neoplasm of colon, rectum, anus and anal canal |
| D13 | D | Neoplasms | Benign neoplasms | Benign neoplasm of other and ill-defined parts of digestive system |
| D14 | D | Neoplasms | Benign neoplasms | Benign neoplasm of middle ear and respiratory system |
| D15 | D | Neoplasms | Benign neoplasms | Benign neoplasm of other and unspecified intrathoracic organs |
| D16 | D | Neoplasms | Benign neoplasms | Benign neoplasm of bone and articular cartilage |
| D17 | D | Neoplasms | Benign neoplasms | Benign lipomatous neoplasm |
| D18 | D | Neoplasms | Benign neoplasms | Haemangioma and lymphangioma, any site |
| D19 | D | Neoplasms | Benign neoplasms | Benign neoplasm of mesothelial tissue |
| D20 | D | Neoplasms | Benign neoplasms | Benign neoplasm of soft tissue of retroperitoneum and peritoneum |
| D21 | D | Neoplasms | Benign neoplasms | Other benign neoplasms of connective and other soft tissue |
| D22 | D | Neoplasms | Benign neoplasms | Melanocytic naevi |
| D23 | D | Neoplasms | Benign neoplasms | Other benign neoplasms of skin |
| D24 | D | Neoplasms | Benign neoplasms | Benign neoplasm of breast |
| D25 | D | Neoplasms | Benign neoplasms | Leiomyoma of uterus |
| D26 | D | Neoplasms | Benign neoplasms | Other benign neoplasms of uterus |
| D27 | D | Neoplasms | Benign neoplasms | Benign neoplasm of ovary |
| D28 | D | Neoplasms | Benign neoplasms | Benign neoplasm of other and unspecified female genital organs |
| D29 | D | Neoplasms | Benign neoplasms | Benign neoplasm of male genital organs |
| D30 | D | Neoplasms | Benign neoplasms | Benign neoplasm of urinary organs |
| D31 | D | Neoplasms | Benign neoplasms | Benign neoplasm of eye and adnexa |
| D32 | D | Neoplasms | Benign neoplasms | Benign neoplasm of meninges |
| D33 | D | Neoplasms | Benign neoplasms | Benign neoplasm of brain and other parts of central nervous system |
| D34 | D | Neoplasms | Benign neoplasms | Benign neoplasm of thyroid gland |
| D35 | D | Neoplasms | Benign neoplasms | Benign neoplasm of other and unspecified endocrine glands |
| D36 | D | Neoplasms | Benign neoplasms | Benign neoplasm of other and unspecified sites |
| D37 | D | Neoplasms | Neoplasms of uncertain or unknown behaviour | Neoplasm of uncertain or unknown behaviour of oral cavity and digestive organs |
| D38 | D | Neoplasms | Neoplasms of uncertain or unknown behaviour | Neoplasm of uncertain or unknown behaviour of middle ear and respiratory and intrathoracic organs |
| D39 | D | Neoplasms | Neoplasms of uncertain or unknown behaviour | Neoplasm of uncertain or unknown behaviour of female genital organs |
| D3A | D | Neoplasms | Neoplasms of uncertain or unknown behaviour | Benign neuroendocrine tumors |
| D40 | D | Neoplasms | Neoplasms of uncertain or unknown behaviour | Neoplasm of uncertain or unknown behaviour of male genital organs |
| D41 | D | Neoplasms | Neoplasms of uncertain or unknown behaviour | Neoplasm of uncertain or unknown behaviour of urinary organs |
| D42 | D | Neoplasms | Neoplasms of uncertain or unknown behaviour | Neoplasm of uncertain or unknown behaviour of meninges |
| D43 | D | Neoplasms | Neoplasms of uncertain or unknown behaviour | Neoplasm of uncertain or unknown behaviour of brain and central nervous system |
| D44 | D | Neoplasms | Neoplasms of uncertain or unknown behaviour | Neoplasm of uncertain or unknown behaviour of endocrine glands |
| D45 | D | Neoplasms | Neoplasms of uncertain or unknown behaviour | Polycythaemia vera |
| D46 | D | Neoplasms | Neoplasms of uncertain or unknown behaviour | Myelodysplastic syndromes |
| D47 | D | Neoplasms | Neoplasms of uncertain or unknown behaviour | Other neoplasms of uncertain or unknown behaviour of lymphoid, haematopoietic and related tissue |
| D48 | D | Neoplasms | Neoplasms of uncertain or unknown behaviour | Neoplasm of uncertain or unknown behaviour of other and unspecified sites |
| D49 | D | Neoplasms | Neoplasms of uncertain or unknown behaviour | Neoplasm of unspecified behavior of digestive system |
| E11 | E | Endocrine, nutritional, and metabolic diseases | Diabetes mellitus | Type 2 diabetes mellitus |
| E70 | E | Endocrine, nutritional, and metabolic diseases | Metabolic disorders | Disorders of aromatic amino-acid metabolism |
| E71 | E | Endocrine, nutritional, and metabolic diseases | Metabolic disorders | Disorders of branched-chain amino-acid metabolism and fatty-acid metabolism |
| E72 | E | Endocrine, nutritional, and metabolic diseases | Metabolic disorders | Other disorders of amino-acid metabolism |
| E73 | E | Endocrine, nutritional, and metabolic diseases | Metabolic disorders | Lactose intolerance |
| E74 | E | Endocrine, nutritional, and metabolic diseases | Metabolic disorders | Other disorders of carbohydrate metabolism |
| E75 | E | Endocrine, nutritional, and metabolic diseases | Metabolic disorders | Disorders of sphingolipid metabolism and other lipid storage disorders |
| E76 | E | Endocrine, nutritional, and metabolic diseases | Metabolic disorders | Disorders of glycosaminoglycan metabolism |
| E77 | E | Endocrine, nutritional, and metabolic diseases | Metabolic disorders | Disorders of glycoprotein metabolism |
| E78 | E | Endocrine, nutritional, and metabolic diseases | Metabolic disorders | Disorders of lipoprotein metabolism and other lipidaemias |
| E79 | E | Endocrine, nutritional, and metabolic diseases | Metabolic disorders | Disorders of purine and pyrimidine metabolism |
| E80 | E | Endocrine, nutritional, and metabolic diseases | Metabolic disorders | Disorders of porphyrin and bilirubin metabolism |
| E83 | E | Endocrine, nutritional, and metabolic diseases | Metabolic disorders | Disorders of mineral metabolism |
| E84 | E | Endocrine, nutritional, and metabolic diseases | Metabolic disorders | Cystic fibrosis |
| E85 | E | Endocrine, nutritional, and metabolic diseases | Metabolic disorders | Amyloidosis |
| E86 | E | Endocrine, nutritional, and metabolic diseases | Metabolic disorders | Volume depletion |
| E87 | E | Endocrine, nutritional, and metabolic diseases | Metabolic disorders | Other disorders of fluid, electrolyte and acid-base balance |
| E88 | E | Endocrine, nutritional, and metabolic diseases | Metabolic disorders | Other metabolic disorders |
| G20 | G | Diseases of the nervous system | Extrapyramidal and movement disorders | Parkinson disease |
| G21 | G | Diseases of the nervous system | Extrapyramidal and movement disorders | Secondary parkinsonism |
| G23 | G | Diseases of the nervous system | Extrapyramidal and movement disorders | Other degenerative diseases of basal ganglia |
| G24 | G | Diseases of the nervous system | Extrapyramidal and movement disorders | Dystonia |
| G25 | G | Diseases of the nervous system | Extrapyramidal and movement disorders | Other extrapyramidal and movement disorders |
| G30 | G | Diseases of the nervous system | Other degenerative diseases of the nervous system | Alzheimer disease |
| G31 | G | Diseases of the nervous system | Other degenerative diseases of the nervous system | Other degenerative diseases of nervous system, not elsewhere classified |
| I10 | I | Diseases of the circulatory system | Hypertensive diseases | Essential (primary) hypertension |
| I11 | I | Diseases of the circulatory system | Hypertensive diseases | Hypertensive heart disease |
| I12 | I | Diseases of the circulatory system | Hypertensive diseases | Hypertensive renal disease |
| I13 | I | Diseases of the circulatory system | Hypertensive diseases | Hypertensive heart and renal disease |
| I15 | I | Diseases of the circulatory system | Hypertensive diseases | Secondary hypertension |
| I20 | I | Diseases of the circulatory system | Ischaemic heart diseases | Angina pectoris |
| I21 | I | Diseases of the circulatory system | Ischaemic heart diseases | Acute myocardial infarction |
| I22 | I | Diseases of the circulatory system | Ischaemic heart diseases | Subsequent myocardial infarction |
| I23 | I | Diseases of the circulatory system | Ischaemic heart diseases | Certain current complications following acute myocardial infarction |
| I24 | I | Diseases of the circulatory system | Ischaemic heart diseases | Other acute ischaemic heart diseases |
| I25 | I | Diseases of the circulatory system | Ischaemic heart diseases | Chronic ischaemic heart disease |
| I30 | I | Diseases of the circulatory system | Other forms of heart disease | Acute pericarditis |
| I31 | I | Diseases of the circulatory system | Other forms of heart disease | Other diseases of pericardium |
| I33 | I | Diseases of the circulatory system | Other forms of heart disease | Acute and subacute endocarditis |
| I34 | I | Diseases of the circulatory system | Other forms of heart disease | Nonrheumatic mitral valve disorders |
| I35 | I | Diseases of the circulatory system | Other forms of heart disease | Nonrheumatic aortic valve disorders |
| I36 | I | Diseases of the circulatory system | Other forms of heart disease | Nonrheumatic tricuspid valve disorders |
| I37 | I | Diseases of the circulatory system | Other forms of heart disease | Pulmonary valve disorders |
| I38 | I | Diseases of the circulatory system | Other forms of heart disease | Endocarditis, valve unspecified |
| I40 | I | Diseases of the circulatory system | Other forms of heart disease | Acute myocarditis |
| I42 | I | Diseases of the circulatory system | Other forms of heart disease | Cardiomyopathy |
| I44 | I | Diseases of the circulatory system | Other forms of heart disease | Atrioventricular and left bundle-branch block |
| I45 | I | Diseases of the circulatory system | Other forms of heart disease | Other conduction disorders |
| I46 | I | Diseases of the circulatory system | Other forms of heart disease | Cardiac arrest |
| I47 | I | Diseases of the circulatory system | Other forms of heart disease | Paroxysmal tachycardia |
| I48 | I | Diseases of the circulatory system | Other forms of heart disease | Atrial fibrillation and flutter |
| I49 | I | Diseases of the circulatory system | Other forms of heart disease | Other cardiac arrhythmias |
| I50 | I | Diseases of the circulatory system | Other forms of heart disease | Heart failure |
| I51 | I | Diseases of the circulatory system | Other forms of heart disease | Complications and ill-defined descriptions of heart disease |
| I60 | I | Diseases of the circulatory system | Cerebrovascular diseases | Subarachnoid haemorrhage |
| I61 | I | Diseases of the circulatory system | Cerebrovascular diseases | Intracerebral haemorrhage |
| I62 | I | Diseases of the circulatory system | Cerebrovascular diseases | Other nontraumatic intracranial haemorrhage |
| I63 | I | Diseases of the circulatory system | Cerebrovascular diseases | Cerebral infarction |
| I65 | I | Diseases of the circulatory system | Cerebrovascular diseases | Occlusion and stenosis of precerebral arteries, not resulting in cerebral infarction |
| I66 | I | Diseases of the circulatory system | Cerebrovascular diseases | Occlusion and stenosis of cerebral arteries, not resulting in cerebral infarction |
| I67 | I | Diseases of the circulatory system | Cerebrovascular diseases | Other cerebrovascular diseases |
| I69 | I | Diseases of the circulatory system | Cerebrovascular diseases | Sequelae of cerebrovascular disease |
| I70 | I | Diseases of the circulatory system | Diseases of arteries, arterioles and capillaries | Atherosclerosis |
| I71 | I | Diseases of the circulatory system | Diseases of arteries, arterioles and capillaries | Aortic aneurysm and dissection |
| I72 | I | Diseases of the circulatory system | Diseases of arteries, arterioles and capillaries | Other aneurysm and dissection |
| I73 | I | Diseases of the circulatory system | Diseases of arteries, arterioles and capillaries | Other peripheral vascular diseases |
| I74 | I | Diseases of the circulatory system | Diseases of arteries, arterioles and capillaries | Arterial embolism and thrombosis |
| I75 | I | Diseases of the circulatory system | Diseases of arteries, arterioles and capillaries | Atheroembolism of lower extremity |
| I77 | I | Diseases of the circulatory system | Diseases of arteries, arterioles and capillaries | Other disorders of arteries and arterioles |
| I78 | I | Diseases of the circulatory system | Diseases of arteries, arterioles and capillaries | Diseases of capillaries |
| I80 | I | Diseases of the circulatory system | Diseases of veins, lymphatic vessels and lymph nodes, not elsewhere classified | Phlebitis and thrombophlebitis |
| I81 | I | Diseases of the circulatory system | Diseases of veins, lymphatic vessels and lymph nodes, not elsewhere classified | Portal vein thrombosis |
| I82 | I | Diseases of the circulatory system | Diseases of veins, lymphatic vessels and lymph nodes, not elsewhere classified | Other venous embolism and thrombosis |
| I83 | I | Diseases of the circulatory system | Diseases of veins, lymphatic vessels and lymph nodes, not elsewhere classified | Varicose veins of lower extremities |
| I85 | I | Diseases of the circulatory system | Diseases of veins, lymphatic vessels and lymph nodes, not elsewhere classified | Oesophageal varices |
| I86 | I | Diseases of the circulatory system | Diseases of veins, lymphatic vessels and lymph nodes, not elsewhere classified | Varicose veins of other sites |
| I87 | I | Diseases of the circulatory system | Diseases of veins, lymphatic vessels and lymph nodes, not elsewhere classified | Other disorders of veins |
| I88 | I | Diseases of the circulatory system | Diseases of veins, lymphatic vessels and lymph nodes, not elsewhere classified | Nonspecific lymphadenitis |
| I89 | I | Diseases of the circulatory system | Diseases of veins, lymphatic vessels and lymph nodes, not elsewhere classified | Other noninfective disorders of lymphatic vessels and lymph nodes |
| J40 | J | Diseases of the respiratory system | Chronic lower respiratory diseases | Bronchitis, not specified as acute or chronic |
| J41 | J | Diseases of the respiratory system | Chronic lower respiratory diseases | Simple and mucopurulent chronic bronchitis |
| J42 | J | Diseases of the respiratory system | Chronic lower respiratory diseases | Unspecified chronic bronchitis |
| J43 | J | Diseases of the respiratory system | Chronic lower respiratory diseases | Emphysema |
| J44 | J | Diseases of the respiratory system | Chronic lower respiratory diseases | Other chronic obstructive pulmonary disease |
| J45 | J | Diseases of the respiratory system | Chronic lower respiratory diseases | Asthma |
| J47 | J | Diseases of the respiratory system | Chronic lower respiratory diseases | Bronchiectasis |
| K20 | K | Diseases of the digestive system | Diseases of oesophagus, stomach and duodenum | Oesophagitis |
| K21 | K | Diseases of the digestive system | Diseases of oesophagus, stomach and duodenum | Gastro-oesophageal reflux disease |
| K22 | K | Diseases of the digestive system | Diseases of oesophagus, stomach and duodenum | Other diseases of oesophagus |
| K25 | K | Diseases of the digestive system | Diseases of oesophagus, stomach and duodenum | Gastric ulcer |
| K26 | K | Diseases of the digestive system | Diseases of oesophagus, stomach and duodenum | Duodenal ulcer |
| K27 | K | Diseases of the digestive system | Diseases of oesophagus, stomach and duodenum | Peptic ulcer, site unspecified |
| K28 | K | Diseases of the digestive system | Diseases of oesophagus, stomach and duodenum | Gastrojejunal ulcer |
| K29 | K | Diseases of the digestive system | Diseases of oesophagus, stomach and duodenum | Gastritis and duodenitis |
| K30 | K | Diseases of the digestive system | Diseases of oesophagus, stomach and duodenum | Functional dyspepsia |
| K31 | K | Diseases of the digestive system | Diseases of oesophagus, stomach and duodenum | Other diseases of stomach and duodenum |
| K40 | K | Diseases of the digestive system | Hernia | Inguinal hernia |
| K41 | K | Diseases of the digestive system | Hernia | Femoral hernia |
| K42 | K | Diseases of the digestive system | Hernia | Umbilical hernia |
| K43 | K | Diseases of the digestive system | Hernia | Ventral hernia |
| K44 | K | Diseases of the digestive system | Hernia | Diaphragmatic hernia |
| K45 | K | Diseases of the digestive system | Hernia | Other abdominal hernia |
| K46 | K | Diseases of the digestive system | Hernia | Unspecified abdominal hernia |
| K50 | K | Diseases of the digestive system | Noninfective enteritis and colitis | Crohn disease [regional enteritis] |
| K51 | K | Diseases of the digestive system | Noninfective enteritis and colitis | Ulcerative colitis |
| K52 | K | Diseases of the digestive system | Noninfective enteritis and colitis | Other noninfective gastroenteritis and colitis |
| K55 | K | Diseases of the digestive system | Other diseases of intestines | Vascular disorders of intestine |
| K56 | K | Diseases of the digestive system | Other diseases of intestines | Paralytic ileus and intestinal obstruction without hernia |
| K57 | K | Diseases of the digestive system | Other diseases of intestines | Diverticular disease of intestine |
| K58 | K | Diseases of the digestive system | Other diseases of intestines | Irritable bowel syndrome |
| K59 | K | Diseases of the digestive system | Other diseases of intestines | Other functional intestinal disorders |
| K60 | K | Diseases of the digestive system | Other diseases of intestines | Fissure and fistula of anal and rectal regions |
| K61 | K | Diseases of the digestive system | Other diseases of intestines | Abscess of anal and rectal regions |
| K62 | K | Diseases of the digestive system | Other diseases of intestines | Other diseases of anus and rectum |
| K63 | K | Diseases of the digestive system | Other diseases of intestines | Other diseases of intestine |
| K64 | K | Diseases of the digestive system | Other diseases of intestines | Haemorrhoids and perianal venous thrombosis |
| K71 | K | Diseases of the digestive system | Diseases of liver | Toxic liver disease |
| K72 | K | Diseases of the digestive system | Diseases of liver | Hepatic failure, not elsewhere classified |
| K73 | K | Diseases of the digestive system | Diseases of liver | Chronic hepatitis, not elsewhere classified |
| K74 | K | Diseases of the digestive system | Diseases of liver | Fibrosis and cirrhosis of liver |
| K75 | K | Diseases of the digestive system | Diseases of liver | Other inflammatory liver diseases |
| K76 | K | Diseases of the digestive system | Diseases of liver | Other diseases of liver |
| K80 | K | Diseases of the digestive system | Disorders of gallbladder, biliary tract and pancreas | Cholelithiasis |
| K81 | K | Diseases of the digestive system | Disorders of gallbladder, biliary tract and pancreas | Cholecystitis |
| K82 | K | Diseases of the digestive system | Disorders of gallbladder, biliary tract and pancreas | Other diseases of gallbladder |
| K83 | K | Diseases of the digestive system | Disorders of gallbladder, biliary tract and pancreas | Other diseases of biliary tract |
| K85 | K | Diseases of the digestive system | Disorders of gallbladder, biliary tract and pancreas | Acute pancreatitis |
| K86 | K | Diseases of the digestive system | Disorders of gallbladder, biliary tract and pancreas | Other diseases of pancreas |
| K90 | K | Diseases of the digestive system | Other diseases of the digestive system | Intestinal malabsorption |
| K91 | K | Diseases of the digestive system | Other diseases of the digestive system | Postprocedural disorders of digestive system, not elsewhere classified |
| K92 | K | Diseases of the digestive system | Other diseases of the digestive system | Other diseases of digestive system |
| M02 | M | Diseases of the musculoskeletal system and connective tissue | Arthropathies | Reactive arthropathies |
| M04 | M | Diseases of the musculoskeletal system and connective tissue | Arthropathies | Autoinflammatory syndromes |
| M05 | M | Diseases of the musculoskeletal system and connective tissue | Arthropathies | Seropositive rheumatoid arthritis |
| M06 | M | Diseases of the musculoskeletal system and connective tissue | Arthropathies | Other rheumatoid arthritis |
| M07 | M | Diseases of the musculoskeletal system and connective tissue | Arthropathies | Psoriatic and enteropathic arthropathies |
| M10 | M | Diseases of the musculoskeletal system and connective tissue | Arthropathies | Gout |
| M11 | M | Diseases of the musculoskeletal system and connective tissue | Arthropathies | Other crystal arthropathies |
| M12 | M | Diseases of the musculoskeletal system and connective tissue | Arthropathies | Other specific arthropathies |
| M13 | M | Diseases of the musculoskeletal system and connective tissue | Arthropathies | Other arthritis |
| M15 | M | Diseases of the musculoskeletal system and connective tissue | Arthropathies | Polyarthrosis |
| M16 | M | Diseases of the musculoskeletal system and connective tissue | Arthropathies | Coxarthrosis [arthrosis of hip] |
| M17 | M | Diseases of the musculoskeletal system and connective tissue | Arthropathies | Gonarthrosis [arthrosis of knee] |
| M18 | M | Diseases of the musculoskeletal system and connective tissue | Arthropathies | Arthrosis of first carpometacarpal joint |
| M19 | M | Diseases of the musculoskeletal system and connective tissue | Arthropathies | Other arthrosis |
| M1A | M | Diseases of the musculoskeletal system and connective tissue | Arthropathies | Chronic gout |
| M22 | M | Diseases of the musculoskeletal system and connective tissue | Arthropathies | Disorders of patella |
| M23 | M | Diseases of the musculoskeletal system and connective tissue | Arthropathies | Internal derangement of knee |
| M24 | M | Diseases of the musculoskeletal system and connective tissue | Arthropathies | Other specific joint derangements |
| M25 | M | Diseases of the musculoskeletal system and connective tissue | Arthropathies | Other joint disorders, not elsewhere classified |
| M80 | M | Diseases of the musculoskeletal system and connective tissue | Osteopathies and chondropathies | Osteoporosis with pathological fracture |
| M81 | M | Diseases of the musculoskeletal system and connective tissue | Osteopathies and chondropathies | Osteoporosis without pathological fracture |
| M83 | M | Diseases of the musculoskeletal system and connective tissue | Osteopathies and chondropathies | Adult osteomalacia |
| M84 | M | Diseases of the musculoskeletal system and connective tissue | Osteopathies and chondropathies | Disorders of continuity of bone |
| M85 | M | Diseases of the musculoskeletal system and connective tissue | Osteopathies and chondropathies | Other disorders of bone density and structure |
| M86 | M | Diseases of the musculoskeletal system and connective tissue | Osteopathies and chondropathies | Osteomyelitis |
| M87 | M | Diseases of the musculoskeletal system and connective tissue | Osteopathies and chondropathies | Osteonecrosis |
| M88 | M | Diseases of the musculoskeletal system and connective tissue | Osteopathies and chondropathies | Paget disease of bone [osteitis deformans] |
| M89 | M | Diseases of the musculoskeletal system and connective tissue | Osteopathies and chondropathies | Other disorders of bone |
| M93 | M | Diseases of the musculoskeletal system and connective tissue | Osteopathies and chondropathies | Other osteochondropathies |
| M94 | M | Diseases of the musculoskeletal system and connective tissue | Osteopathies and chondropathies | Other disorders of cartilage |
| N18 | N | Diseases of the genitourinary system | Renal failure | Chronic kidney disease |
