# Supplementary material for: Human lifespan and sex-specific patterns of resilience to disease: a retrospective population-wide cohort study
Source: BMC Med. 2024 Jan 8;22:17. doi: 10.1186/s12916-023-03206-w (PMC10773063; doi:10.1186/s12916-023-03206-w)
Supplement: Supplementary file 5 — Additional file 5. Hazard ratios (HR) for multisystem involvement in different biological systems from the main ICD-10 categories by age of death and sex. [file 12916_2023_3206_MOESM5_ESM.docx]

Table S2. Hazard ratios (HR) for multisystem involvement in different biological systems from the main ICD-10 categories by age of death and sex.

| Number of systems affected | HR (95% CI) for Age of death | HR (95% CI) for Sex (Women) | HR (95% CI) for Age of death:Sex (Women) |
| --- | --- | --- | --- |
| 2 | 0.862 (0.862-0.863) | 1.032 (1.025-1.038) | 1.001 (1.000-1.002) |
| 3 | 0.849 (0.848-0.849) | 1.027 (1.020-1.033) | 1.001 (1.000-1.001) |
| 4 | 0.830 (0.829-0.831) | 1.002 (0.994-1.009) | 0.998 (0.997-0.999) |
| 5 | 0.804 (0.803-0.805) | 0.968 (0.959-0.977) | 0.995 (0.994-0.996) |
| 6 | 0.768 (0.767-0.770) | 0.909 (0.895-0.922) | 0.993 (0.991-0.995) |
| 7 | 0.727 (0.723-0.731) | 0.846 (0.821-0.872) | 0.990 (0.986-0.994) |
| 8 | 0.692 (0.679-0.705) | 0.863 (0.782-0.951 | 0.979 (0.966-0.992) |

*HR: Hazard ratio, CI: Confidence interval*
