# Supplementary material for: Human lifespan and sex-specific patterns of resilience to disease: a retrospective population-wide cohort study
Source: BMC Med. 2024 Jan 8;22:17. doi: 10.1186/s12916-023-03206-w (PMC10773063; doi:10.1186/s12916-023-03206-w)
Supplement: Supplementary file 6 — Additional file 6. Prevalence of escapers according to ICD-10 categories, by sex and decade of death. [file 12916_2023_3206_MOESM6_ESM.docx]

Table S3. Prevalence of escapers according to ICD-10 categories, by sex and decade of death

| Pathology | All | | 50-59 | | 60-69 | | 70-79 | | 80-89 | | 90-99 | | 100-105+ | |
| --- | --- | --- | --- | --- | --- | --- | --- | --- | --- | --- | --- | --- | --- | --- |
|  | Women (N=243127) | Men (N=238931) | Women (N=5224) | Men (N=10328) | Women (N=13648) | Men (N=30224) | Women (N=35106) | Men (N=60357) | Women (N=103706) | Men (N=97017) | Women (N=80457) | Men (N=39712) | Women (N=4986) | Men (N=1293) |
| Neoplasms | 64.3% [64.1%;64.5%] | 48.7% [48.5%;48.9%] | 31.5% [30.3%;32.8%] | 45.3% [44.3%;46.3%] | 36.3% [35.5%;37.2%] | 40.1% [39.6%;40.7%] | 50.9% [50.4%;51.4%] | 43.5% [43.1%;43.9%] | 65.4% [65.1%;65.7%] | 50.8% [50.5%;51.2%] | 74.4% [74.1%;74.7%] | 58.4% [58.0%;58.9%] | 81.7% [80.6%;82.7%] | 68.0% [65.4%;70.5%] |
| Malignant neoplasms | 75.4% [75.2%;75.6%] | 59.6% [59.4%;59.8%] | 41.4% [40.1%;42.8%] | 52.0% [51.1%;53.0%] | 46.8% [45.9%;47.6%] | 48.3% [47.7%;48.9%] | 62.3% [61.8%;62.8%] | 53.3% [52.9%;53.7%] | 77.1% [76.9%;77.4%] | 62.6% [62.3%;63.0%] | 85.1% [84.8%;85.3%] | 71.5% [71.0%;71.9%] | 90.1% [89.2%;90.9%] | 80.0% [77.8%;82.2%] |
| In situ neoplasms | 97.7% [97.6%;97.7%] | 96.4% [96.3%;96.5%] | 96.5% [95.9%;97.0%] | 98.0% [97.7%;98.3%] | 97.4% [97.2%;97.7%] | 97.7% [97.5%;97.8%] | 97.7% [97.5%;97.8%] | 96.8% [96.7%;96.9%] | 97.8% [97.7%;97.9%] | 96.0% [95.9%;96.1%] | 97.6% [97.5%;97.7%] | 95.5% [95.3%;95.7%] | 97.5% [97.1%;97.9%] | 96.3% [95.1%;97.3%] |
| Benign neoplasms | 87.3% [87.2%;87.4%] | 85.4% [85.2%;85.5%] | 78.1% [76.9%;79.2%] | 87.6% [87.0%;88.3%] | 78.1% [77.4%;78.8%] | 84.4% [83.9%;84.8%] | 83.3% [82.9%;83.7%] | 84.1% [83.8%;84.4%] | 87.3% [87.1%;87.5%] | 85.5% [85.3%;85.7%] | 90.7% [90.5%;90.9%] | 87.1% [86.8%;87.4%] | 93.9% [93.2%;94.6%] | 88.6% [86.7%;90.2%] |
| Neoplasms of uncertain or unknown behaviour | 97.2% [97.1%;97.2%] | 96.8% [96.7%;96.8%] | 97.3% [96.8%;97.7%] | 98.3% [98.0%;98.5%] | 97.3% [97.0%;97.6%] | 97.6% [97.4%;97.8%] | 97.0% [96.8%;97.2%] | 97.0% [96.9%;97.1%] | 97.0% [96.9%;97.1%] | 96.3% [96.2%;96.4%] | 97.4% [97.2%;97.5%] | 96.5% [96.3%;96.7%] | 98.2% [97.8%;98.6%] | 97.3% [96.3%;98.1%] |
| Endocrine, nutritional and metabolic diseases | 32.7% [32.5%;32.8%] | 32.6% [32.4%;32.8%] | 53.5% [52.1%;54.8%] | 43.9% [42.9%;44.8%] | 34.4% [33.6%;35.2%] | 31.1% [30.6%;31.6%] | 26.5% [26.1%;27.0%] | 27.3% [27.0%;27.7%] | 29.2% [28.9%;29.5%] | 31.5% [31.2%;31.8%] | 37.0% [36.7%;37.3%] | 40.7% [40.2%;41.2%] | 50.9% [49.5%;52.3%] | 56.9% [54.2%;59.6%] |
| Diabetes mellitus | 67.2% [67.0%;67.4%] | 62.4% [62.2%;62.6%] | 82.0% [81.0%;83.1%] | 72.0% [71.1%;72.9%] | 70.1% [69.4%;70.9%] | 61.1% [60.5%;61.6%] | 60.1% [59.6%;60.6%] | 56.8% [56.4%;57.2%] | 63.5% [63.2%;63.8%] | 61.5% [61.1%;61.8%] | 72.8% [72.5%;73.1%] | 71.4% [70.9%;71.8%] | 82.2% [81.1%;83.2%] | 81.7% [79.5%;83.8%] |
| Metabolic disorders | 44.9% [44.7%;45.1%] | 47.3% [47.1%;47.5%] | 61.6% [60.2%;62.9%] | 55.9% [54.9%;56.8%] | 45.6% [44.8%;46.4%] | 45.4% [44.9%;46.0%] | 40.7% [40.2%;41.2%] | 42.9% [42.5%;43.3%] | 42.3% [42.0%;42.6%] | 46.7% [46.4%;47.1%] | 48.0% [47.7%;48.4%] | 53.6% [53.1%;54.1%] | 59.9% [58.5%;61.3%] | 67.9% [65.3%;70.4%] |
| Diseases of the nervous system | 73.0% [72.8%;73.2%] | 81.3% [81.1%;81.5%] | 97.1% [96.6%;97.6%] | 97.9% [97.6%;98.1%] | 92.4% [91.9%;92.8%] | 94.7% [94.4%;94.9%] | 80.2% [79.8%;80.6%] | 85.8% [85.5%;86.0%] | 68.6% [68.3%;68.9%] | 75.0% [74.7%;75.3%] | 70.3% [70.0%;70.6%] | 75.3% [74.9%;75.7%] | 79.6% [78.5%;80.7%] | 82.6% [80.4%;84.6%] |
| Extrapyramidal and movement disorders | 92.6% [92.5%;92.7%] | 92.7% [92.6%;92.8%] | 98.6% [98.3%;98.9%] | 99.0% [98.8%;99.2%] | 96.5% [96.2%;96.8%] | 97.3% [97.1%;97.5%] | 92.3% [92.0%;92.6%] | 93.3% [93.1%;93.5%] | 91.2% [91.1%;91.4%] | 90.3% [90.1%;90.5%] | 93.2% [93.0%;93.4%] | 92.4% [92.1%;92.6%] | 95.5% [94.9%;96.0%] | 95.2% [93.9%;96.3%] |
| Other degenerative diseases of the nervous system | 78.5% [78.4%;78.7%] | 87.0% [86.9%;87.1%] | 98.4% [98.0%;98.7%] | 98.8% [98.6%;99.0%] | 95.3% [95.0%;95.7%] | 97.1% [96.9%;97.2%] | 86.3% [86.0%;86.7%] | 91.1% [90.9%;91.3%] | 75.0% [74.7%;75.2%] | 82.4% [82.1%;82.6%] | 75.3% [75.0%;75.6%] | 81.3% [80.9%;81.7%] | 83.5% [82.5%;84.5%] | 86.2% [84.2%;88.1%] |
| Diseases of the circulatory system | 10.6% [10.5%;10.7%] | 13.3% [13.2%;13.5%] | 49.4% [48.0%;50.8%] | 39.7% [38.7%;40.6%] | 29.0% [28.2%;29.8%] | 24.0% [23.5%;24.5%] | 13.6% [13.2%;13.9%] | 13.7% [13.5%;14.0%] | 7.96% [7.79%;8.12%] | 9.09% [8.91%;9.28%] | 7.08% [6.90%;7.26%] | 8.15% [7.88%;8.42%] | 9.93% [9.11%;10.8%] | 13.5% [11.6%;15.4%] |
| Hypertensive diseases | 23.3% [23.2%;23.5%] | 32.2% [32.0%;32.4%] | 68.4% [67.1%;69.6%] | 59.9% [58.9%;60.8%] | 48.2% [47.3%;49.0%] | 43.6% [43.0%;44.2%] | 28.0% [27.5%;28.5%] | 32.1% [31.7%;32.5%] | 19.9% [19.6%;20.1%] | 27.6% [27.4%;27.9%] | 18.7% [18.4%;19.0%] | 27.7% [27.3%;28.1%] | 22.2% [21.0%;23.4%] | 34.9% [32.3%;37.5%] |
| Ischaemic heart diseases | 86.7% [86.5%;86.8%] | 77.7% [77.5%;77.9%] | 96.4% [95.9%;96.9%] | 88.6% [88.0%;89.2%] | 93.4% [93.0%;93.8%] | 82.4% [82.0%;82.8%] | 87.7% [87.3%;88.0%] | 77.0% [76.7%;77.4%] | 85.4% [85.2%;85.6%] | 75.4% [75.1%;75.7%] | 86.1% [85.9%;86.4%] | 77.7% [77.3%;78.2%] | 87.4% [86.5%;88.3%] | 84.7% [82.6%;86.6%] |
| Other forms of heart disease | 51.7% [51.5%;51.9%] | 52.3% [52.1%;52.5%] | 88.8% [87.9%;89.6%] | 83.2% [82.5%;83.9%] | 78.2% [77.5%;78.9%] | 72.3% [71.8%;72.8%] | 61.1% [60.6%;61.6%] | 58.2% [57.8%;58.6%] | 47.8% [47.4%;48.1%] | 44.2% [43.9%;44.5%] | 45.7% [45.4%;46.1%] | 39.8% [39.3%;40.3%] | 54.1% [52.7%;55.5%] | 48.8% [46.0%;51.6%] |
| Cerebrovascular diseases | 82.8% [82.7%;83.0%] | 80.7% [80.6%;80.9%] | 95.4% [94.8%;95.9%] | 92.6% [92.1%;93.1%] | 91.8% [91.3%;92.3%] | 87.9% [87.5%;88.2%] | 85.5% [85.1%;85.9%] | 81.6% [81.3%;81.9%] | 81.3% [81.0%;81.5%] | 77.7% [77.4%;77.9%] | 81.3% [81.0%;81.5%] | 78.3% [77.8%;78.7%] | 83.8% [82.7%;84.8%] | 82.8% [80.6%;84.8%] |
| Diseases of arteries arterioles and capillaries | 91.9% [91.7%;92.0%] | 82.3% [82.1%;82.4%] | 95.8% [95.2%;96.3%] | 91.2% [90.6%;91.7%] | 93.9% [93.4%;94.3%] | 84.1% [83.7%;84.6%] | 92.2% [91.9%;92.5%] | 80.4% [80.0%;80.7%] | 91.3% [91.1%;91.5%] | 80.9% [80.6%;81.1%] | 91.7% [91.5%;91.9%] | 84.7% [84.3%;85.0%] | 93.9% [93.2%;94.5%] | 88.9% [87.0%;90.5%] |
| Diseases of veins lymphatic vessels and lymph nodes not elsewhere classified | 70.5% [70.3%;70.6%] | 83.8% [83.7%;84.0%] | 81.8% [80.7%;82.8%] | 86.6% [85.9%;87.2%] | 75.3% [74.6%;76.0%] | 85.5% [85.1%;85.9%] | 70.9% [70.4%;71.4%] | 84.5% [84.2%;84.8%] | 69.4% [69.1%;69.6%] | 83.4% [83.2%;83.6%] | 69.9% [69.6%;70.2%] | 82.0% [81.6%;82.4%] | 74.4% [73.2%;75.7%] | 82.9% [80.7%;84.9%] |
| Diseases of the respiratory system | 75.4% [75.3%;75.6%] | 62.4% [62.2%;62.6%] | 81.4% [80.3%;82.4%] | 79.1% [78.3%;79.9%] | 76.6% [75.9%;77.3%] | 68.4% [67.9%;69.0%] | 75.4% [74.9%;75.8%] | 60.7% [60.3%;61.0%] | 74.8% [74.6%;75.1%] | 59.6% [59.3%;59.9%] | 75.5% [75.2%;75.8%] | 62.7% [62.3%;63.2%] | 78.3% [77.2%;79.5%] | 69.1% [66.5%;71.6%] |
| Chronic lower respiratory diseases | 75.4% [75.3%;75.6%] | 62.4% [62.2%;62.6%] | 81.4% [80.3%;82.4%] | 79.1% [78.3%;79.9%] | 76.6% [75.9%;77.3%] | 68.4% [67.9%;69.0%] | 75.4% [74.9%;75.8%] | 60.7% [60.3%;61.0%] | 74.8% [74.6%;75.1%] | 59.6% [59.3%;59.9%] | 75.5% [75.2%;75.8%] | 62.7% [62.3%;63.2%] | 78.3% [77.2%;79.5%] | 69.1% [66.5%;71.6%] |
| Diseases of the digestive system | 38.5% [38.3%;38.7%] | 35.6% [35.5%;35.8%] | 47.3% [45.9%;48.6%] | 45.6% [44.7%;46.6%] | 40.0% [39.2%;40.9%] | 38.5% [38.0%;39.1%] | 39.1% [38.6%;39.6%] | 37.2% [36.8%;37.6%] | 38.0% [37.7%;38.3%] | 34.2% [33.9%;34.5%] | 37.9% [37.6%;38.3%] | 31.9% [31.4%;32.4%] | 40.8% [39.4%;42.1%] | 34.6% [32.0%;37.2%] |
| Diseases of oesophagus stomach and duodenum | 77.4% [77.3%;77.6%] | 75.8% [75.6%;75.9%] | 79.0% [77.9%;80.1%] | 80.3% [79.5%;81.0%] | 74.6% [73.8%;75.3%] | 75.4% [74.9%;75.9%] | 76.3% [75.8%;76.7%] | 75.3% [74.9%;75.6%] | 77.1% [76.8%;77.3%] | 75.6% [75.3%;75.8%] | 78.5% [78.2%;78.8%] | 76.0% [75.5%;76.4%] | 81.5% [80.4%;82.6%] | 77.8% [75.4%;80.0%] |
| Hernia | 85.1% [84.9%;85.2%] | 78.2% [78.1%;78.4%] | 91.6% [90.9%;92.4%] | 86.9% [86.3%;87.6%] | 87.6% [87.0%;88.2%] | 81.9% [81.5%;82.4%] | 85.6% [85.2%;86.0%] | 79.4% [79.1%;79.7%] | 84.3% [84.1%;84.5%] | 76.8% [76.5%;77.1%] | 84.8% [84.5%;85.0%] | 75.0% [74.6%;75.5%] | 88.2% [87.3%;89.1%] | 76.9% [74.5%;79.1%] |
| Noninfective enteritis and colitis | 91.6% [91.5%;91.7%] | 92.0% [91.9%;92.1%] | 93.1% [92.4%;93.8%] | 94.7% [94.2%;95.1%] | 92.9% [92.5%;93.3%] | 93.7% [93.4%;93.9%] | 92.1% [91.8%;92.4%] | 92.7% [92.5%;92.9%] | 91.5% [91.3%;91.7%] | 91.3% [91.1%;91.4%] | 91.3% [91.1%;91.4%] | 90.7% [90.4%;91.0%] | 92.5% [91.8%;93.2%] | 89.6% [87.8%;91.2%] |
| Other diseases of intestines | 65.3% [65.1%;65.5%] | 67.3% [67.1%;67.5%] | 77.0% [75.9%;78.2%] | 78.9% [78.1%;79.7%] | 71.1% [70.3%;71.8%] | 73.9% [73.4%;74.4%] | 68.4% [67.9%;68.9%] | 69.9% [69.5%;70.2%] | 64.7% [64.4%;65.0%] | 64.8% [64.5%;65.1%] | 63.1% [62.7%;63.4%] | 61.8% [61.3%;62.2%] | 63.2% [61.8%;64.5%] | 62.4% [59.7%;65.1%] |
| Diseases of liver | 94.6% [94.5%;94.7%] | 91.9% [91.8%;92.1%] | 89.5% [88.7%;90.3%] | 83.8% [83.0%;84.5%] | 88.5% [88.0%;89.1%] | 85.3% [84.8%;85.6%] | 90.3% [90.0%;90.6%] | 89.7% [89.5%;89.9%] | 94.7% [94.5%;94.8%] | 94.2% [94.1%;94.4%] | 97.4% [97.3%;97.5%] | 96.8% [96.7%;97.0%] | 98.9% [98.5%;99.1%] | 98.9% [98.2%;99.4%] |
| Disorders of gallbladder biliary tract and pancreas | 85.3% [85.1%;85.4%] | 87.4% [87.3%;87.6%] | 89.2% [88.4%;90.1%] | 91.0% [90.4%;91.5%] | 86.6% [86.0%;87.2%] | 89.6% [89.2%;89.9%] | 85.3% [84.9%;85.6%] | 88.4% [88.2%;88.7%] | 84.9% [84.7%;85.1%] | 86.5% [86.3%;86.7%] | 85.2% [84.9%;85.4%] | 85.7% [85.3%;86.0%] | 87.0% [86.0%;87.9%] | 86.9% [85.0%;88.7%] |
| Other diseases of the digestive system | 95.5% [95.4%;95.6%] | 94.5% [94.4%;94.6%] | 97.2% [96.8%;97.7%] | 95.3% [94.9%;95.7%] | 96.2% [95.9%;96.6%] | 95.2% [95.0%;95.5%] | 95.9% [95.7%;96.1%] | 95.0% [94.8%;95.2%] | 95.4% [95.3%;95.6%] | 94.3% [94.2%;94.4%] | 95.3% [95.1%;95.4%] | 93.6% [93.4%;93.9%] | 95.4% [94.8%;96.0%] | 93.6% [92.1%;94.9%] |
| Diseases of the musculoskeletal system and connective tissue | 31.5% [31.3%;31.6%] | 49.7% [49.5%;49.9%] | 59.2% [57.8%;60.5%] | 70.2% [69.3%;71.1%] | 41.7% [40.9%;42.5%] | 59.8% [59.3%;60.4%] | 33.0% [32.5%;33.5%] | 52.8% [52.4%;53.2%] | 29.2% [28.9%;29.4%] | 45.6% [45.3%;46.0%] | 29.9% [29.6%;30.2%] | 41.8% [41.3%;42.3%] | 36.1% [34.8%;37.5%] | 46.7% [44.0%;49.5%] |
| Arthropathies | 39.4% [39.2%;39.6%] | 52.3% [52.1%;52.5%] | 65.2% [63.9%;66.5%] | 72.2% [71.4%;73.1%] | 51.1% [50.3%;52.0%] | 62.2% [61.7%;62.8%] | 42.5% [42.0%;43.0%] | 55.4% [55.0%;55.8%] | 37.2% [36.9%;37.5%] | 48.5% [48.2%;48.8%] | 36.9% [36.6%;37.3%] | 44.4% [43.9%;44.9%] | 41.7% [40.4%;43.1%] | 49.2% [46.4%;52.0%] |
| Osteopathies and chondropathies | 72.0% [71.9%;72.2%] | 93.2% [93.1%;93.3%] | 88.6% [87.7%;89.5%] | 96.3% [95.9%;96.7%] | 77.6% [76.9%;78.3%] | 94.9% [94.7%;95.2%] | 71.0% [70.6%;71.5%] | 93.8% [93.6%;94.0%] | 69.7% [69.4%;69.9%] | 92.4% [92.2%;92.6%] | 73.0% [72.7%;73.3%] | 92.2% [91.9%;92.5%] | 81.1% [80.0%;82.1%] | 94.4% [93.0%;95.6%] |
| Diseases of the genitourinary system | 71.1% [70.9%;71.3%] | 74.2% [74.1%;74.4%] | 95.3% [94.7%;95.8%] | 94.8% [94.4%;95.2%] | 90.4% [89.9%;90.9%] | 89.5% [89.1%;89.8%] | 80.7% [80.3%;81.1%] | 79.7% [79.4%;80.0%] | 69.9% [69.6%;70.2%] | 68.8% [68.5%;69.1%] | 63.8% [63.5%;64.2%] | 62.7% [62.2%;63.2%] | 66.5% [65.2%;67.8%] | 65.1% [62.5%;67.7%] |
| Renal failure | 71.1% [70.9%;71.3%] | 74.2% [74.1%;74.4%] | 95.3% [94.7%;95.8%] | 94.8% [94.4%;95.2%] | 90.4% [89.9%;90.9%] | 89.5% [89.1%;89.8%] | 80.7% [80.3%;81.1%] | 79.7% [79.4%;80.0%] | 69.9% [69.6%;70.2%] | 68.8% [68.5%;69.1%] | 63.8% [63.5%;64.2%] | 62.7% [62.2%;63.2%] | 66.5% [65.2%;67.8%] | 65.1% [62.5%;67.7%] |

*The specific diseases belonging to each category can be found in Supplementary Table 1.*
